# All coffee types decrease the risk of adverse clinical outcomes in chronic liver disease: a UK Biobank study

**DOI:** 10.1186/s12889-021-10991-7

**Published:** 2021-06-22

**Authors:** Oliver J. Kennedy, Jonathan A. Fallowfield, Robin Poole, Peter C. Hayes, Julie Parkes, Paul J. Roderick

**Affiliations:** 1 Primary Care & Population Sciences, Faculty of Medicine, University of Southampton, Southampton, SO17 1BJ UK; 2 University of Edinburgh Centre for Inflammation Research, Queen’s Medical Research Institute, Edinburgh Bioquarter, Edinburgh, EH16 4TJ UK

**Keywords:** Coffee, Chronic liver disease, Cirrhosis, Hepatocellular carcinoma

## Abstract

**Background:**

Chronic liver disease (CLD) is a growing cause of morbidity and mortality worldwide, particularly in low to middle-income countries with high disease burden and limited treatment availability. Coffee consumption has been linked with lower rates of CLD, but little is known about the effects of different coffee types, which vary in chemical composition. This study aimed to investigate associations of coffee consumption, including decaffeinated, instant and ground coffee, with chronic liver disease outcomes.

**Methods:**

A total of 494,585 UK Biobank participants with known coffee consumption and electronic linkage to hospital, death and cancer records were included in this study. Cox regression was used to estimate hazard ratios (HR) of incident CLD, incident CLD or steatosis, incident hepatocellular carcinoma (HCC) and death from CLD according to coffee consumption of any type as well as for decaffeinated, instant and ground coffee individually.

**Results:**

Among 384,818 coffee drinkers and 109,767 non-coffee drinkers, there were 3600 cases of CLD, 5439 cases of CLD or steatosis, 184 cases of HCC and 301 deaths from CLD during a median follow-up of 10.7 years. Compared to non-coffee drinkers, coffee drinkers had lower adjusted HRs of CLD (HR 0.79, 95% CI 0.72–0.86), CLD or steatosis (HR 0.80, 95% CI 0.75–0.86), death from CLD (HR 0.51, 95% CI 0.39–0.67) and HCC (HR 0.80, 95% CI 0.54–1.19). The associations for decaffeinated, instant and ground coffee individually were similar to all types combined.

**Conclusion:**

The finding that all types of coffee are protective against CLD is significant given the increasing incidence of CLD worldwide and the potential of coffee as an intervention to prevent CLD onset or progression.

**Supplementary Information:**

The online version contains supplementary material available at 10.1186/s12889-021-10991-7.

## Background

Chronic liver disease (CLD) is a major health problem worldwide. Between 1990 and 2017, global deaths due to CLD increased from 899,000 (1.9% of total) to 1.32 million (2.4% of total) [1]. During the same period, disability-adjusted life-years lost to CLD increased from 30.5 million to 41.4 million. The burden of CLD is highest in low to middle-income countries where treatment options are also limited. Sub-Saharan Africa is the region that is most affected followed by Central and South America, Eastern Europe and Southeast Asia [1, 2]. The commonest aetiologies of CLD are alcohol-related liver disease (ALD), chronic hepatitis B and C infection, and non-alcoholic fatty liver disease (NAFLD) [3]. These conditions involve destruction and regeneration of liver parenchyma leading to liver fibrosis and then cirrhosis. Cirrhosis can be fatal due to complications related to portal hypertension, liver failure or the development of hepatocellular carcinoma (HCC).

Coffee is a popular beverage in most societies [4]. It comprises hundreds of chemical compounds, some of which are thought to have in vivo properties, including caffeine, chlorogenic acid, kahweol and cafestol [5]. Observational and laboratory studies suggest that consumption of coffee confers a protective effect against CLD, including cirrhosis and HCC [6–8]. This effect has been observed among drinkers of caffeinated and, to a lesser extent, decaffeinated coffee [9]. Based on these observations, coffee has been proposed as a potential intervention to prevent CLD onset and progression or HCC in at-risk patients [7]. However, the attributes of an effective coffee-based intervention remain uncertain in terms of quantity and preparation, which substantially affects composition (e.g. decaffeinated coffee lacks caffeine, while filtered and instant coffee have only minimal amounts of kahweol and cafestol) [10]. The aim of this study was to investigate associations of coffee consumption, including the effects of different coffee types (and, thus, composition), with CLD outcomes in a large prospective cohort.

## Methods

### Study population and baseline assessment

UK Biobank is a prospective longitudinal study aimed at identifying genetic and behavioural determinants of health outcomes. A protocol for the study is available online [11]. Prospective participants were identified from National Health Service registers, which include most of the UK population. However, invitations were mostly sent to people within 10 miles of the ~ 22 UK Biobank assessment centres, which were in urban areas. Around 5 million invitations were sent with 500,000 men and women aged 40–69 between 2006 and 2010 agreeing to participate. All participants attended an assessment centre, where they answered questions about medical history and lifestyle, underwent physical examination, and gave urine and blood samples.

Baseline coffee consumption was ascertained by a touchscreen questionnaire. Participants were asked how many cups of coffee they drank each day. Responses of > 10 required confirmation and > 99 were rejected. Coffee drinkers were asked what type of coffee they usually drank from “decaffeinated”, “instant”, “ground (including espresso)” or “other”. Participants were also asked whether they had been told by a doctor that they had diabetes and whether they currently or previously smoked tobacco. The response rates to these questions were > 99%.

Participants were asked how frequently they consumed alcohol (number of days each week or month) and about weekly / monthly quantities of alcohol types (e.g. spirits, red / white wine, beer, cider etc.). To standardise responses, the questionnaire specified portion sizes for each type of alcohol (e.g. “there are six glasses in an average bottle [of wine]”). After extracting the data from UK Biobank, weekly “units” (one unit is equivalent to 10 mL of pure ethanol) were calculated for each participant using the specified portion sizes and assumed alcohol content, as shown in supplementary Table 1. While 99% of participants stated their frequency of alcohol consumption, only 83.7% stated quantities that enabled estimation of total weekly units. The baseline characteristics of participants according to whether weekly units could be estimated are shown in supplementary Table 2, with participants without weekly units more likely to be female, deprived, non-smokers, consume less coffee, have diabetes, and drink alcohol less than once a week (i.e. “special occasions only” or “one to three times a month”). Participants without unit data had marginally higher rates of incident CLD (0.8% vs. 0.7%) and incident CLD or steatosis (1.3% vs. 1.1%), a marginally lower rate of death from CLD (0.045% vs 0.064%), and a similar rate of incident HCC (0.037% vs. 0.037%).

Participants’ body mass indices (BMI) were calculated from height and weight measured by trained nurses during the initial assessment centre visit. For these measurements, participants removed shoes and heavy clothing. Height was measured to the nearest centimetre and weight to the nearest 0.5 kg.

UK Biobank assigned a Townsend deprivation score to each participant based on postcode of habitation. Townsend deprivation scores are calculated from levels of employment, home and car ownership and household overcrowding in a given area [12]. The median Townsend deprivation score was − 2.1, which is less deprived than the UK average of − 0.35 and comparable to the 25th percentile of UK deprivation of − 2.35 [13].

All UK Biobank participants gave consent for continued follow-up by linkage to electronic health records. These included death records and cancer registers, which are maintained by the Office for National Statistics and the Registrar General’s Office, and hospital records, which are held by the Department of Health’s Hospital Episode Statistics and the Scottish Morbidity Record. Ethics approval was given by North West Multi-Centre Research Ethics Committee.

### Inclusion and exclusion criteria

The UK Biobank dataset used in this study included 502,528 participants. Participants were excluded who withdrew consent (*n* = 22) or had a baseline medical condition that likely represented CLD (*n* = 5691). Baseline conditions were identified from hospital and cancer records and from interviews during the baseline assessment centre visit. Lists of ICD (International Classification of Diseases) codes and conditions from the baseline interview used to exclude participants are shown in supplementary Tables 3a-c. Participants were also excluded if they did not provide baseline coffee consumption (*n* = 2230, 0.44% of total). Supplementary Table 4 shows that these participants were more likely to be male, of non-white ethnicity (58.7% vs. 5.4%), deprived and have diabetes, but less likely to drink alcohol or smoke compared to the participants who did provide baseline coffee consumption. After the exclusions, there were 494,585 participants (~ 98% of total) available for inclusion in this study (the exact numbers used in each analysis are specified below).

### Outcome ascertainment

The main outcomes were incident CLD, incident HCC and death from CLD. The term “CLD” was taken to include chronic liver diseases involving underlying destruction of liver parenchyma and fibrosis. This included HCC, which was also analysed as a separate outcome. However, CLD did not include simple fatty liver disease (i.e. isolated steatosis without inflammation). The effect of not including steatosis was examined by analysing a fourth outcome of “incident CLD or steatosis”.

The outcomes of incident CLD, incident CLD or steatosis and HCC were identified from hospital records, cancer registers and the primary or secondary causes on death records using the linked datasets described above. The outcome of death from CLD was identified from the primary causes on death records. The ICD-9 and ICD-10 codes used to identify cases are shown in Table 1.

**Table 1:** ICD-9 and ICD-10 codes used to identify outcomes in hospital records, cancer registers and death records

	ICD-9	ICD-10
**CLD**	1550, 4560–4562, 5710, 5712–5714, 57,150–57,152, 57,158, 57,159, 5718, 5719, 5723, 5724, 5728, 7895	C22.0, K70.0-K70.4, K70.9, K72.1, K72.9, K73.0-K73.2, K73.8, K73.9, K74.0-K74.2, K74.6, K75.8, K75.9, K76.6, K76.7, R18, I85.0, I85.9, I86.4, I98.2, I98.3
**Steatosis**	5718	K76.0
**HCC**	1550	C22.0

Abbreviations: *ICD-9* International Classification of Diseases 9th Revision), *ICD-10* (International Classification of Diseases 10th Revision), *CLD* (chronic liver disease), *HCC* (hepatocellular carcinoma)

### Statistical analyses

Cox proportional hazard regression was used to estimate age and sex adjusted associations of confounding variables with incident CLD and death from CLD. Non-cases were right censored at the date of loss to follow-up, date of non-CLD death or the end of follow-up. The date of loss to follow-up was recorded by UK Biobank and based on when it stopped receiving follow-up data for a participant. The end of follow-up was the date of data extraction (November 2019). The confounding variables considered were based on previous similar studies, known risk factors for CLD and informed using causal directed acyclic graphs [7, 14]. These were Townsend deprivation, smoking status (never, current or previous), diabetes (yes or no), BMI, ethnicity (white or other ethnicity), alcohol frequency (less than weekly, once or twice a week, three or four times a week, and daily or almost daily) and weekly alcohol units.

Cox proportional hazard regression was used to estimate unadjusted and adjusted hazard ratios (HRs) with 95% confidence intervals (CI) for each outcome according to consumption of coffee of all types combined as well as for decaffeinated, instant and ground (including espresso) coffee separately. Non-drinkers of coffee were used as the reference group in all analyses. HRs of each outcome were calculated according to any amount of coffee compared to none as well as for 0.5, 1, 2, 3, 4 and ≥ 5 cups each day compared to none.

For the adjusted analyses, which are described in further detail below, it was necessary to determine if the confounding variables that were continuous (i.e. age, BMI, deprivation and weekly alcohol units) were linearly or non-linearly associated with the outcomes. For this purpose, univariate Cox hazard regression was performed using penalised splines [15]. Where the associations were non-linear, as determined by a Wald-type test using the nonlinear coefficient estimates [16], flexible splines were used in further analyses. Otherwise, linear terms were used. This approach is similar to previous studies that have modelled continuous variables [15].

Adjusted HRs were calculated using three different Cox models. The first model included covariates for age and sex only. The second additionally included covariates for Townsend deprivation, smoking status, diabetes, ethnicity, alcohol frequency and BMI. The third model was the same as the second but included weekly alcohol units instead of alcohol frequency. All the analyses were performed with complete cases, which equated to 494,585 (100% of total), 494,585 (100%), 486,251 (98.3%) and 408,835 (82.6%) participants, respectively, in the unadjusted and three adjusted models described above.

Potential violations of the proportional hazards (PH) assumption were investigated by examining, for each variable, correlations between scaled Schoenfeld residuals and time. Where there was an indication of a violation, log(−log) and cumulative incidence plots were constructed and visually inspected, and sensitivity analyses were performed that involved stratification on the variable of interest. Stratification accounts for violation of the PH assumption by allowing a different baseline hazard in each strata.

To investigate possible reverse causation, HRs were calculated for each outcome according to coffee consumption after removing all cases occurring within the first 5 years from recruitment. Finally, subgroup analyses were performed by calculating adjusted HRs of CLD in each BMI category (i.e. < 25, 25–29, ≥30 kg/m^2^) and in participants with and without diabetes. All analyses were performed in R (Version 4.0.3). The Strengthening the Reporting of Observational Studies in Epidemiology (STROBE) guidelines [17] were followed in reporting this study.

### Patient involvement

This work was informed by the results of a patient and public involvement focus group and survey of patients with CLD. This demonstrated a high level of interest among patients in potential randomised controlled trials evaluating coffee as an intervention in CLD.

## Results

**Fig. 1:**
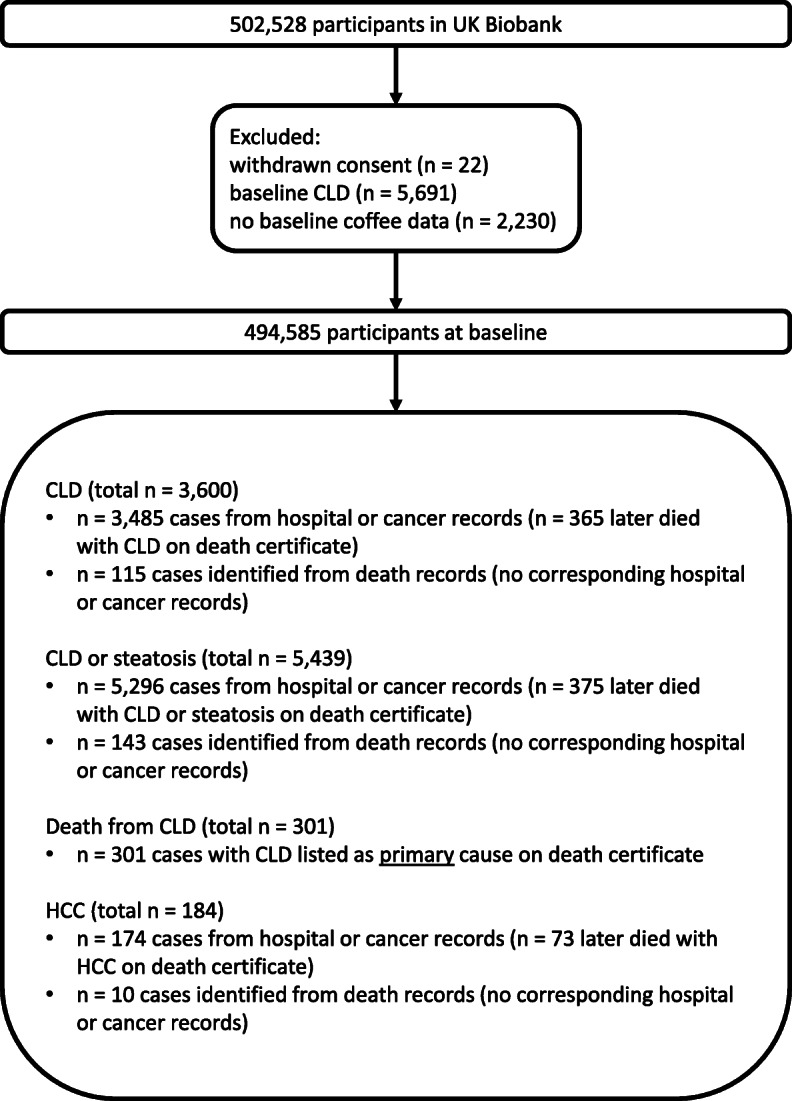
A flow diagram showing the derivation of the cohort used in this study and the sources for identification of cases for each outcome

A total of 494,585 participants with known baseline coffee consumption and without baseline CLD were followed-up for a median of 10.7 years (interquartile range: 1.4 years). During the follow-up, there were 3600 cases of incident CLD, 5439 cases of incident CLD or steatosis, 184 cases of incident HCC (including 83 deaths) and 301 deaths from CLD (Fig. 1).

There were 384,818 (78%) coffee drinkers (all types combined), with a median consumption of 2 cups each day (interquartile range = 3 cups each day), in addition to 109,767 (22%) non-coffee drinkers. Non-coffee drinkers were more likely to be non-smokers and teetotal but were more deprived and had a higher prevalence of diabetes and obesity (Table 2). Coffee drinkers in the highest category of consumption (i.e. ≥5 cups each day) were most likely to be male, overweight and smoke.

**Table 2:** Baseline characteristics of the 494,585 participants in the UK Biobank cohort and distribution of incident chronic liver disease, incident chronic liver disease or steatosis, death from chronic liver disease and incident hepatocellular carcinoma according to coffee consumption

Coffee (cups/d)	0	Half	1	2	3	4	≥5
N	109,767	35,876	99,179	92,886	60,479	41,515	54,883
Median age (years) at baseline (IQR)	56 (13)	57 (13)	59 (13)	59 (13)	58 (12)	58 (13)	57 (13)
Sex (% female)	58.3	55.3	57.2	54.3	52.4	50	47.5
**Ethnicity (%)**							
White	89.7	92.7	93.8	95.7	96.8	97.4	97.6
Other ethnicity	9.9	7.0	5.9	4.0	2.9	2.2	2.0
Unknown	0.4	0.4	0.3	0.3	0.3	0.4	0.3
Median Townsend deprivation score	−1.70	−2.15	−2.23	−2.32	−2.38	−2.35	−2.06
**Alcohol frequency (%)**							
Never	14.4	6.9	6.4	5.3	5.1	5.5	7.7
Special occasions only	15.9	12.1	11.1	9.1	8.8	9.2	12.0
One to three times a month	12.6	12.7	11.0	9.9	9.8	10.1	11.8
Once or twice a week	24.9	26.6	27.0	26.1	25.3	25.8	25.4
Three or four times a week	17.6	23.2	24.0	25.8	26.3	25.8	22.3
Daily or almost daily	14.5	18.5	20.4	23.6	24.6	23.5	20.7
Unknown	0.1	0.1	0.1	0.1	0.1	0.1	0.1
Mean weekly units (IQR)	11 (24.3)	15 (24.8)	15.2 (23.3)	17.5 (23.8)	18.2 (24.2)	18.8 (25.2)	17.5 (27.8)
**Diabetes (%)**							
Yes	5.7	5.0	4.9	4.7	5.0	5.3	5.7
No	93.9	94.7	94.8	95.0	94.8	94.4	94.0
Unknown	0.4	0.4	0.3	0.2	0.2	0.3	0.3
**Median BMI in kg/m**^**2**^ **(IQR)**	26.8 (6.1)	26.4 (5.7)	26.3 (5.6)	26.5 (5.5)	26.8 (5.5)	27.2 (5.7)	27.5 (5.9)
**Smoking (%)**							
Never	58.6	58.3	57.6	55.3	53.6	50.3	42.3
Previous	31.3	33.1	34.8	35.7	36.2	36.9	35.9
Current	9.7	8.2	7.3	8.7	9.9	12.4	21.3
Unknown	0.4	0.4	0.3	0.4	0.4	0.4	0.4
**CLD cases (n)**							
Total (%)	927 (0.84)	281 (0.78)	707 (0.71)	651 (0.70)	380 (0.63)	267 (0.64)	387 (0.71)
First 5 years excluded (%)	449 (0.41)	134 (0.37)	329 (0.33)	320 (0.34)	185 (0.31)	122 (0.29)	213 (0.39)
**CLD or steatosis cases (n)**							
Total (%)	1412 (1.29)	394 (1.10)	1063 (1.07)	965 (1.04)	564 (0.93)	400 (0.96)	641 (1.17)
First 5 years excluded (%)	724 (0.66)	198 (0.55)	521 (0.53)	497 (0.54)	289 (0.48)	197 (0.47)	357 (0.65)
**HCC cases (n)**							
Total (%)	44 (0.04)	10 (0.03)	33 (0.03)	40 (0.04)	24 (0.04)	15 (0.04)	18 (0.03)
First 5 years excluded (%)	28 (0.03)	1 (< 0.01)	3 (< 0.01)	3 (< 0.01)	1 (< 0.01)	0 (0.00)	3 (0.01)
**CLD deaths(n)**							
Total (%)	97 (0.09)	23 (0.06)	49 (0.05)	56 (0.06)	29 (0.05)	17 (0.04)	30 (0.05)
First 5 years excluded (%)	59 (0.05)	12 (0.03)	30 (0.03)	41 (0.04)	20 (0.03)	9 (0.02)	21 (0.04)

Abbreviations: *N* (number), *BMI* (body mass index), *IQR* (interquartile range), *CLD* (chronic liver disease), *HCC* (hepatocellular carcinoma)

Of coffee drinkers, 73,644 (19%), 212,586 (55%) and 86,987 (23%), respectively, were drinkers of decaffeinated, instant and ground (including espresso) coffee. The baseline characteristics of these participants are shown in supplementary Tables 5, 6, 7, 8. Decaffeinated coffee drinkers were more likely to be female (63.1% vs. 50.5% for instant and 51.7% for ground), older (median age at baseline 59 vs. 58 and 57) and less likely to be current smokers (6.7% vs 12.6 and 9.2%). Drinkers of ground coffee had the highest median weekly alcohol consumption (20.2 units vs. 17.2 for instant and 12.8 for decaffeinated) and were least likely to have diabetes (3.9% vs. 5.4 and 5.0%).

Townsend deprivation quartile, previous or current smoking, diabetes and BMI were associated with increased risk of incident CLD and death from CLD, as shown in supplementary Table 9. The highest quartile of weekly alcohol units and daily or almost daily alcohol consumption were also associated with increased risk but not the lower categories. There was no association of ethnicity with incident CLD or death from CLD. The results of the univariate Cox analyses using penalised splines are shown in in supplementary Figs. 1, 2, 3, 4. The tests for non-linearity were significant for BMI (*p*-value < 0.001) and weekly alcohol units (*p*-value < 0.001) but not for age (*p*-value 0.74) or deprivation (*p*-value 0.19). Thus, these continuous covariates were modelled in the main analysis using splines for BMI and weekly alcohol units and linear terms for age and deprivation.

**Fig. 2:**
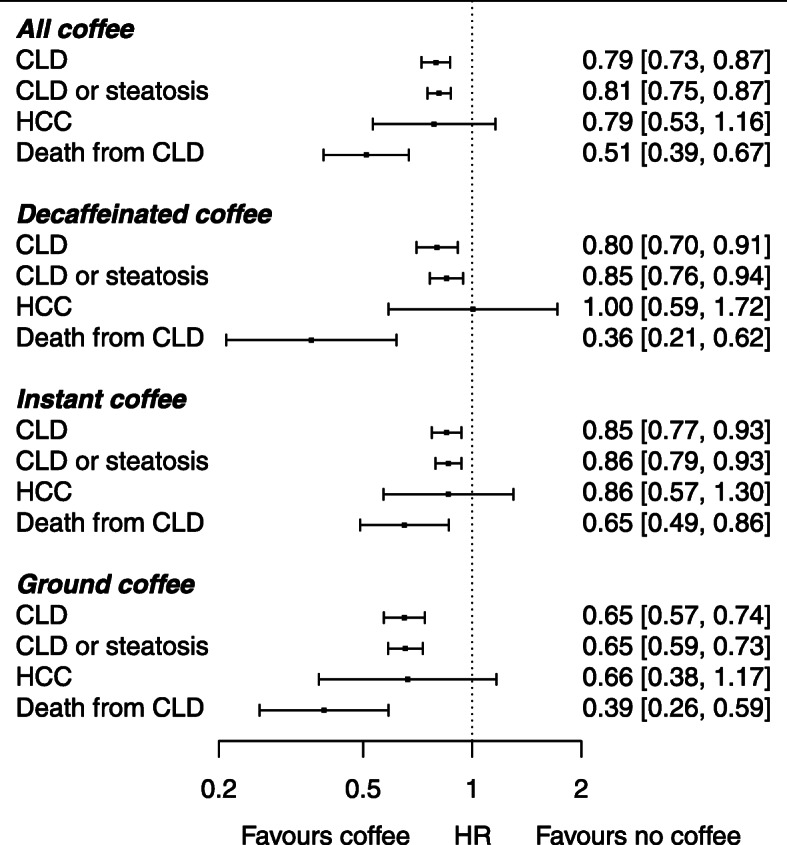
A forest plot showing the associations between consumption of all coffee, decaffeinated coffee, instant coffee and ground coffee (including espresso) with incident CLD, incident CLD or steatosis, incident HCC and death from CLD

Coffee drinkers (all types combined) had lower fully adjusted risks than non-coffee drinkers of incident CLD (HR 0.79, 95% CI 0.72–0.86), incident CLD or steatosis (HR 0.80, 95% CI 0.75–0.86), and death from CLD (HR 0.51, 95% CI 0.39–0.67) (Table 3 and Fig. 2). There was a similar, though less statistically significant, association between coffee consumption and incident HCC (HR 0.80, 95% CI 0.54–1.19). The reductions in HRs were proportional to the quantity of coffee consumption up to around 3–4 cups each day beyond which further increases in consumption provided no additional benefit. The adjusted HRs were very similar though slightly larger in magnitude compared to the unadjusted HRs. After excluding cases occurring within the first 5 years, coffee drinkers still had lower fully adjusted risks than non-coffee drinkers of incident CLD (HR 0.79, 95% CI 0.70–0.90), incident CLD or steatosis (HR 0.79, 95% CI 0.71–0.88) and death from CLD (HR 0.56, 95% CI 0.39–0.78).

**Table 3:** Hazard ratios with 95% confidence intervals for associations of coffee consumption (all types) with incident chronic liver disease, incident chronic liver disease or steatosis, death from chronic liver disease and incident hepatocellular carcinoma, as calculated in unadjusted and multivariate analyses

	HR^**1**^ (***n*** = 494,585)	HR^**2**^ (***n*** = 494,585)	HR^**3**^ (***n*** = 486,251)	HR^**4**^ (***n*** = 408,835)
**CLD**				
No coffee	1 (ref.)	1 (ref.)	1 (ref.)	1 (ref.)
0.5 cups/d	0.93 (0.81–1.06)	0.88 (0.77–1.01)	0.92 (0.80–1.06)	0.90 (0.76–1.05)
1 cup/d	0.84 (0.76–0.93)	0.78 (0.70–0.86)	0.84 (0.76–0.94)	0.87 (0.77–0.97)
2 cups/d	0.83 (0.75–0.92)	0.76 (0.69–0.84)	0.81 (0.73–0.90)	0.80 (0.71–0.90)
3 cups/d	0.74 (0.66–0.84)	0.68 (0.60–0.77)	0.70 (0.62–0.80)	0.73 (0.64–0.83)
4 cups/d	0.76 (0.66–0.87)	0.70 (0.61–0.80)	0.70 (0.60–0.80)	0.71 (0.61–0.82)
≥ 5 cups/d	0.84 (0.74–0.94)	0.79 (0.71–0.89)	0.70 (0.62–0.79)	0.72 (0.63–0.83)
Any amount	0.82 (0.76–0.88)	0.76 (0.71–0.82)	0.78 (0.72–0.84)	0.79 (0.72–0.86)
Any amount (excluding cases within first 5 years)	0.83 (0.74–0.92)	0.77 (0.69–0.86)	0.79 (0.70–0.88)	0.79 (0.70–0.90)
**CLD or steatosis**				
No coffee	1 (ref.)	1 (ref.)	1 (ref.)	1 (ref.)
0.5 cups/d	0.85 (0.76–0.95)	0.82 (0.73–0.92)	0.90 (0.80–1.01)	0.87 (0.76–1.00)
1 cup/d	0.83 (0.77–0.90)	0.78 (0.72–0.85)	0.89 (0.81–0.96)	0.88 (0.80–0.97)
2 cups/d	0.81 (0.74–0.87)	0.75 (0.69–0.82)	0.84 (0.77–0.92)	0.81 (0.74–0.89)
3 cups/d	0.72 (0.66–0.80)	0.68 (0.61–0.75)	0.73 (0.66–0.81)	0.74 (0.66–0.83)
4 cups/d	0.75 (0.67–0.84)	0.70 (0.63–0.78)	0.71 (0.64–0.80)	0.71 (0.62–0.81)
≥ 5 cups/d	0.91 (0.83–1.00)	0.87 (0.80–0.96)	0.79 (0.71–0.87)	0.78 (0.70–0.87)
Any amount	0.81 (0.76–0.86)	0.77 (0.72–0.81)	0.82 (0.76–0.87)	0.80 (0.75–0.86)
Any amount (excluding cases within first 5 years)	0.81 (0.74–0.88)	0.77 (0.71–0.84)	0.81 (0.74–0.89)	0.79 (0.71–0.88)
**HCC**				
No coffee	1 (ref.)	1 (ref.)	1 (ref.)	1 (ref.)
0.5 cups/d	0.69 (0.35–1.38)	0.63 (0.32–1.25)	0.63 (0.31–1.30)	0.51 (0.22–1.23)
1 cup/d	0.83 (0.53–1.30)	0.71 (0.45–1.12)	0.75 (0.47–1.21)	0.70 (0.41–1.19)
2 cups/d	1.07 (0.70–1.64)	0.90 (0.59–1.38)	0.91 (0.58–1.43)	1.03 (0.63–1.66)
3 cups/d	0.99 (0.60–1.62)	0.84 (0.51–1.37)	0.87 (0.52–1.44)	1.02 (0.60–1.74)
4 cups/d	0.90 (0.50–1.62)	0.77 (0.43–1.38)	0.76 (0.42–1.38)	0.74 (0.38–1.43)
≥ 5 cups/d	0.82 (0.47–1.42)	0.75 (0.43–1.29)	0.68 (0.39–1.19)	0.62 (0.33–1.18)
Any amount	0.91 (0.65–1.27)	0.78 (0.56–1.10)	0.79 (0.55–1.12)	0.80 (0.54–1.19)
Any amount (excluding cases within first 5 years)	0.83 (0.54–1.28)	0.71 (0.46–1.10)	0.73 (0.46–1.15)	0.74 (0.45–1.23)
**Death from CLD**				
No coffee	1 (ref.)	1 (ref.)	1 (ref.)	1 (ref.)
0.5 cups/d	0.72 (0.46–1.14)	0.67 (0.42–1.06)	0.69 (0.43–1.10)	0.60 (0.36–1.02)
1 cup/d	0.56 (0.40–0.79)	0.51 (0.36–0.72)	0.54 (0.38–0.76)	0.55 (0.38–0.80)
2 cups/d	0.68 (0.49–0.95)	0.60 (0.43–0.84)	0.58 (0.41–0.81)	0.60 (0.42–0.87)
3 cups/d	0.54 (0.36–0.82)	0.47 (0.31–0.72)	0.45 (0.30–0.69)	0.49 (0.32–0.76)
4 cups/d	0.46 (0.28–0.78)	0.40 (0.24–0.67)	0.36 (0.21–0.61)	0.40 (0.23–0.69)
≥ 5 cups/d	0.62 (0.41–0.93)	0.54 (0.36–0.81)	0.40 (0.26–0.62)	0.39 (0.25–0.63)
Any amount	0.60 (0.47–0.76)	0.53 (0.42–0.68)	0.50 (0.39–0.64)	0.51 (0.39–0.67)
Any amount (excluding cases within first 5 years)	0.64 (0.47–0.87)	0.57 (0.42–0.77)	0.55 (0.40–0.76)	0.56 (0.39–0.78)

Abbreviations: *CLD* (chronic liver disease), *HCC* (hepatocellular carcinoma). ^1^unadjusted, ^2^adjusted for age and sex, ^3^as for ^2^ and additionally adjusted for deprivation (Townsend score), smoking status (never, current or previous), diabetes (yes or no), ethnicity (white or other ethnicity), alcohol frequency (less than weekly, once or twice a week, three or four times a week, and daily or almost daily) and BMI, ^4^as for ^3^ but adjusted for weekly alcohol units instead of alcohol frequency

Schoenfeld residuals from the fully adjusted Cox model indicated that smoking status did not meet the PH assumption (*p*-value 0.02). Log(−log) and cumulative incidence plots, which are shown in supplementary Figs. 5 and 6 respectively, suggested that there was a period around 200 days when the hazard among current smokers was lower than expected, though this quickly self-corrected thereafter. When the Cox model was stratified on smoking, which accounts for violation in the PH assumption, the estimates for the association between coffee and incident CLD were the same as the unstratified analysis (i.e. HR 0.79, 95% CI 0.72–0.86). In addition, the Schoenfeld residuals did not show any evidence of violation when smoking status was re-categorised as smokers vs. non-smokers, and when this was used in the fully adjusted Cox model the association of coffee consumption with CLD was essentially unchanged. As such, the effect of any violation in the PH assumption on the estimates was negligible.

HRs of incident CLD, incident CLD or steatosis, incident HCC and death from CLD according to consumption of decaffeinated, instant and ground (including espresso) coffee are shown in Tables 4, 5, 6 and Fig. 2. For consumption of each coffee type, there were strong inverse associations with incident CLD, incident CLD or steatosis and death from CLD, though precision was lower due to smaller sample sizes. The associations were similar after excluding cases occurring in the first 5 years of follow-up. There was a weaker inverse association of all coffee types with HCC that did not reach significance in the fully adjusted models. The inverse associations between coffee and CLD were similar in the normal, overweight and obese BMI categories and in participants with and without diabetes (supplementary Table 10).

**Table 4:** Hazard ratios with 95% confidence intervals for associations of decaffeinated coffee consumption with incident chronic liver disease, incident chronic liver disease or steatosis, death from chronic liver disease and incident hepatocellular carcinoma

	HR^**1**^	HR^**2**^	HR^**3**^	HR^**4**^
**CLD**				
No coffee	1 (ref.)	1 (ref.)	1 (ref.)	1 (ref.)
0.5 cups/d	0.85 (0.64–1.13)	0.83 (0.62–1.10)	0.90 (0.66–1.23)	0.85 (0.59–1.23)
1 cup/d	0.75 (0.63–0.91)	0.70 (0.58–0.85)	0.81 (0.67–0.98)	0.82 (0.66–1.02)
2 cups/d	0.78 (0.64–0.95)	0.72 (0.59–0.87)	0.81 (0.66–0.99)	0.83 (0.66–1.04)
3 cups/d	0.74 (0.58–0.94)	0.68 (0.53–0.87)	0.71 (0.55–0.91)	0.76 (0.58–1.01)
4 cups/d	0.80 (0.61–1.05)	0.74 (0.57–0.98)	0.81 (0.61–1.06)	0.85 (0.62–1.15)
≥ 5 cups/d	0.75 (0.58–0.95)	0.71 (0.56–0.91)	0.72 (0.56–0.92)	0.72 (0.54–0.97)
Any amount	0.77 (0.69–0.86)	0.72 (0.64–0.80)	0.79 (0.70–0.88)	0.80 (0.70–0.92)
Any amount (excluding cases within first 5 years)	0.83 (0.71–0.97)	0.78 (0.67–0.91)	0.85 (0.72–1.00)	0.86 (0.72–1.04)
**CLD or steatosis**				
No coffee	1 (ref.)	1 (ref.)	1 (ref.)	1 (ref.)
0.5 cups/d	0.79 (0.62–1.01)	0.78 (0.61–0.99)	0.93 (0.72–1.19)	0.84 (0.62–1.15)
1 cup/d	0.75 (0.65–0.88)	0.71 (0.61–0.83)	0.82 (0.70–0.97)	0.81 (0.67–0.97)
2 cups/d	0.80 (0.68–0.93)	0.75 (0.64–0.87)	0.87 (0.74–1.02)	0.86 (0.72–1.03)
3 cups/d	0.75 (0.62–0.91)	0.70 (0.58–0.85)	0.77 (0.62–0.94)	0.83 (0.66–1.04)
4 cups/d	0.79 (0.64–0.99)	0.75 (0.60–0.93)	0.82 (0.66–1.03)	0.86 (0.67–1.11)
≥ 5 cups/d	0.94 (0.78–1.12)	0.90 (0.75–1.08)	0.90 (0.75–1.08)	0.91 (0.74–1.12)
Any amount	0.80 (0.73–0.87)	0.76 (0.69–0.83)	0.85 (0.77–0.93)	0.85 (0.76–0.95)
Any amount (excluding cases within first 5 years)	0.84 (0.74–0.94)	0.79 (0.70–0.90)	0.88 (0.77–1.00)	0.88 (0.76–1.01)
**HCC**				
No coffee	1 (ref.)	1 (ref.)	1 (ref.)	1 (ref.)
0.5 cups/d	NA	NA	NA	NA
1 cup/d	1.15 (0.56–2.36)	1.03 (0.50–2.12)	1.17 (0.57–2.44)	0.83 (0.32–2.14)
2 cups/d	1.14 (0.54–2.43)	0.99 (0.47–2.11)	1.08 (0.50–2.32)	1.23 (0.54–2.80)
3 cups/d	1.34 (0.57–3.14)	1.17 (0.50–2.75)	1.16 (0.49–2.76)	1.48 (0.61–3.55)
4 cups/d	1.23 (0.44–3.43)	1.08 (0.39–3.02)	1.07 (0.38–2.99)	1.01 (0.31–3.30)
≥ 5 cups/d	0.46 (0.11–1.91)	0.44 (0.11–1.80)	0.39 (0.09–1.61)	0.51 (0.12–2.15)
Any amount	1.08 (0.68–1.72)	0.96 (0.60–1.54)	0.99 (0.61–1.61)	1.01 (0.59–1.74)
Any amount (excluding cases within first 5 years)	1.17 (0.66–2.07)	1.04 (0.58–1.85)	1.10 (0.60–2.00)	1.07 (0.54–2.12)
**Death from CLD**				
No coffee	1 (ref.)	1 (ref.)	1 (ref.)	1 (ref.)
0.5 cups/d	0.50 (0.16–1.56)	0.52 (0.16–1.63)	0.67 (0.21–2.12)	0.82 (0.26–2.62)
1 cup/d	0.29 (0.12–0.71)	0.29 (0.12–0.71)	0.37 (0.15–0.92)	0.18 (0.04–0.72)
2 cups/d	0.39 (0.17–0.89)	0.37 (0.16–0.85)	0.44 (0.19–1.02)	0.37 (0.13–1.01)
3 cups/d	0.30 (0.10–0.96)	0.29 (0.09–0.91)	0.32 (0.10–1.01)	0.39 (0.12–1.24)
4 cups/d	0.56 (0.21–1.52)	0.53 (0.19–1.43)	0.59 (0.21–1.60)	0.55 (0.17–1.75)
≥ 5 cups/d	0.21 (0.05–0.85)	0.20 (0.05–0.83)	0.20 (0.05–0.83)	0.27 (0.07–1.09)
Any amount	0.35 (0.22–0.56)	0.34 (0.22–0.54)	0.40 (0.25–0.64)	0.37 (0.22–0.62)
Any amount (excluding cases within first 5 years)	0.30 (0.16–0.56)	0.28 (0.15–0.53)	0.34 (0.18–0.63)	0.27 (0.13–0.58)

Abbreviations: *CLD* (chronic liver disease), *HCC* (hepatocellular carcinoma). ^1^unadjusted, ^2^adjusted for age and sex, ^3^as for ^2^ and additionally adjusted for deprivation (Townsend score), smoking status (never, current or previous), diabetes (yes or no), ethnicity (white or other ethnicity), alcohol frequency (less than weekly, once or twice a week, three or four times a week, and daily or almost daily) and BMI, ^4^as for ^3^ but adjusted for weekly alcohol units instead of alcohol frequency

**Table 5:** Hazard ratios with 95% confidence intervals for associations of instant coffee consumption with incident chronic liver disease, incident chronic liver disease or steatosis, death from chronic liver disease and incident hepatocellular carcinoma

	HR^**1**^	HR^**2**^	HR^**3**^	HR^**4**^
**CLD**				
No coffee	1 (ref.)	1 (ref.)	1 (ref.)	1 (ref.)
0.5 cups/d	1.12 (0.95–1.32)	1.02 (0.87–1.21)	1.03 (0.87–1.23)	1.04 (0.86–1.27)
1 cup/d	0.94 (0.84–1.06)	0.84 (0.75–0.95)	0.91 (0.80–1.02)	0.95 (0.83–1.09)
2 cups/d	0.93 (0.83–1.05)	0.84 (0.75–0.95)	0.88 (0.78–0.99)	0.87 (0.76–1.00)
3 cups/d	0.81 (0.70–0.93)	0.73 (0.63–0.84)	0.75 (0.64–0.86)	0.79 (0.67–0.92)
4 cups/d	0.80 (0.68–0.94)	0.73 (0.62–0.86)	0.71 (0.60–0.84)	0.73 (0.61–0.88)
≥ 5 cups/d	0.88 (0.77–1.01)	0.84 (0.73–0.96)	0.71 (0.62–0.82)	0.74 (0.63–0.87)
Any amount	0.90 (0.83–0.98)	0.83 (0.76–0.89)	0.82 (0.76–0.90)	0.85 (0.77–0.93)
Any amount (excluding cases within first 5 years)	0.90 (0.80–1.01)	0.83 (0.74–0.93)	0.82 (0.73–0.93)	0.84 (0.73–0.96)
**CLD or steatosis**				
No coffee	1 (ref.)	1 (ref.)	1 (ref.)	1 (ref.)
0.5 cups/d	1.01 (0.87–1.16)	0.94 (0.82–1.08)	0.98 (0.84–1.13)	1.00 (0.84–1.18)
1 cup/d	0.93 (0.84–1.02)	0.85 (0.78–0.94)	0.95 (0.86–1.05)	0.97 (0.87–1.08)
2 cups/d	0.90 (0.82–0.99)	0.83 (0.75–0.92)	0.90 (0.81–1.00)	0.88 (0.78–0.98)
3 cups/d	0.79 (0.70–0.88)	0.73 (0.65–0.82)	0.76 (0.67–0.86)	0.78 (0.68–0.89)
4 cups/d	0.78 (0.69–0.89)	0.73 (0.64–0.84)	0.73 (0.63–0.83)	0.72 (0.62–0.84)
≥ 5 cups/d	0.92 (0.82–1.02)	0.88 (0.79–0.98)	0.77 (0.68–0.86)	0.76 (0.67–0.87)
Any amount	0.89 (0.83–0.95)	0.83 (0.77–0.88)	0.85 (0.79–0.91)	0.85 (0.79–0.92)
Any amount (excluding cases within first 5 years)	0.88 (0.81–0.97)	0.83 (0.76–0.91)	0.84 (0.76–0.93)	0.84 (0.75–0.94)
**HCC**				
No coffee	1 (ref.)	1 (ref.)	1 (ref.)	1 (ref.)
0.5 cups/d	1.16 (0.55–2.47)	0.96 (0.45–2.04)	0.88 (0.40–1.98)	0.61 (0.22–1.74)
1 cup/d	0.84 (0.48–1.47)	0.67 (0.38–1.17)	0.64 (0.35–1.16)	0.62 (0.32–1.20)
2 cups/d	1.44 (0.89–2.31)	1.15 (0.72–1.86)	1.10 (0.67–1.81)	1.26 (0.74–2.14)
3 cups/d	1.15 (0.65–2.03)	0.94 (0.53–1.66)	0.92 (0.51–1.65)	1.08 (0.58–2.00)
4 cups/d	0.88 (0.43–1.81)	0.74 (0.36–1.52)	0.70 (0.34–1.44)	0.67 (0.29–1.51)
≥ 5 cups/d	0.83 (0.44–1.57)	0.74 (0.39–1.40)	0.66 (0.34–1.26)	0.56 (0.26–1.21)
Any amount	1.06 (0.74–1.51)	0.87 (0.61–1.25)	0.82 (0.56–1.20)	0.83 (0.55–1.27)
Any amount (excluding cases within first 5 years)	0.94 (0.59–1.49)	0.77 (0.49–1.22)	0.74 (0.45–1.21)	0.75 (0.43–1.30)
**Death from CLD**				
No coffee	1 (ref.)	1 (ref.)	1 (ref.)	1 (ref.)
0.5 cups/d	0.99 (0.57–1.71)	0.85 (0.50–1.47)	0.84 (0.47–1.47)	0.75 (0.40–1.40)
1 cup/d	0.85 (0.59–1.24)	0.74 (0.51–1.08)	0.76 (0.51–1.12)	0.82 (0.55–1.22)
2 cups/d	0.91 (0.63–1.32)	0.78 (0.54–1.13)	0.74 (0.51–1.10)	0.81 (0.54–1.20)
3 cups/d	0.71 (0.45–1.13)	0.61 (0.38–0.97)	0.58 (0.36–0.93)	0.62 (0.38–1.02)
4 cups/d	0.36 (0.17–0.73)	0.30 (0.15–0.62)	0.27 (0.13–0.56)	0.32 (0.16–0.67)
≥ 5 cups/d	0.56 (0.34–0.93)	0.48 (0.29–0.80)	0.38 (0.23–0.63)	0.34 (0.19–0.61)
Any amount	0.75 (0.58–0.97)	0.64 (0.49–0.83)	0.59 (0.45–0.77)	0.62 (0.47–0.83)
Any amount (excluding cases within first 5 years)	0.81 (0.59–1.13)	0.69 (0.50–0.96)	0.66 (0.47–0.93)	0.69 (0.48–0.99)

Abbreviations: *CLD* (chronic liver disease), *HCC* (hepatocellular carcinoma). ^1^unadjusted, ^2^adjusted for age and sex, ^3^as for ^2^ and additionally adjusted for deprivation (Townsend score), smoking status (never, current or previous), diabetes (yes or no), ethnicity (white or other ethnicity), alcohol frequency (less than weekly, once or twice a week, three or four times a week, and daily or almost daily) and BMI, ^4^as for ^3^ but adjusted for weekly alcohol units instead of alcohol frequency

**Table 6:** Hazard ratios with 95% confidence intervals for associations of ground coffee consumption (including espresso) with incident chronic liver disease, incident chronic liver disease or steatosis, death from CLD and incident hepatocellular carcinoma

	HR^**1**^	HR^**2**^	HR^**3**^	HR^**4**^
**CLD**				
No coffee	1 (ref.)	1 (ref.)	1 (ref.)	1 (ref.)
0.5 cups/d	0.59 (0.44–0.78)	0.58 (0.43–0.78)	0.60 (0.44–0.82)	0.64 (0.46–0.88)
1 cup/d	0.69 (0.58–0.82)	0.67 (0.56–0.79)	0.69 (0.58–0.83)	0.74 (0.61–0.90)
2 cups/d	0.66 (0.55–0.79)	0.61 (0.51–0.74)	0.64 (0.53–0.77)	0.65 (0.53–0.79)
3 cups/d	0.59 (0.46–0.75)	0.55 (0.42–0.70)	0.55 (0.43–0.72)	0.55 (0.42–0.73)
4 cups/d	0.59 (0.43–0.83)	0.55 (0.39–0.76)	0.53 (0.37–0.74)	0.52 (0.36–0.75)
≥ 5 cups/d	0.69 (0.50–0.95)	0.65 (0.47–0.90)	0.56 (0.40–0.78)	0.61 (0.43–0.87)
Any amount	0.65 (0.58–0.72)	0.61 (0.55–0.68)	0.62 (0.55–0.70)	0.64 (0.57–0.73)
Any amount (excluding cases within first 5 years)	0.63 (0.54–0.74)	0.60 (0.51–0.70)	0.62 (0.52–0.74)	0.66 (0.55–0.79)
**CLD or steatosis**				
No coffee	1 (ref.)	1 (ref.)	1 (ref.)	1 (ref.)
0.5 cups/d	0.54 (0.42–0.69)	0.53 (0.42–0.68)	0.58 (0.45–0.76)	0.61 (0.46–0.80)
1 cup/d	0.65 (0.57–0.75)	0.63 (0.55–0.73)	0.73 (0.63–0.85)	0.74 (0.63–0.87)
2 cups/d	0.62 (0.54–0.72)	0.59 (0.51–0.68)	0.66 (0.56–0.77)	0.65 (0.55–0.77)
3 cups/d	0.53 (0.42–0.65)	0.49 (0.40–0.61)	0.56 (0.45–0.70)	0.54 (0.43–0.68)
4 cups/d	0.58 (0.44–0.77)	0.55 (0.42–0.72)	0.55 (0.42–0.73)	0.54 (0.40–0.73)
≥ 5 cups/d	0.78 (0.61–0.99)	0.74 (0.58–0.95)	0.69 (0.54–0.89)	0.66 (0.50–0.87)
Any amount	0.62 (0.56–0.67)	0.59 (0.54–0.64)	0.65 (0.59–0.72)	0.64 (0.58–0.72)
Any amount (excluding cases within first 5 years)	0.59 (0.52–0.67)	0.57 (0.50–0.65)	0.63 (0.55–0.73)	0.62 (0.53–0.72)
**HCC**				
No coffee	1 (ref.)	1 (ref.)	1 (ref.)	1 (ref.)
0.5 cups/d	0.51 (0.12–2.12)	0.51 (0.12–2.09)	0.55 (0.13–2.29)	0.67 (0.16–2.81)
1 cup/d	0.68 (0.31–1.51)	0.64 (0.29–1.42)	0.68 (0.30–1.55)	0.84 (0.37–1.93)
2 cups/d	0.40 (0.14–1.12)	0.35 (0.13–0.99)	0.28 (0.08–0.90)	0.35 (0.11–1.15)
3 cups/d	0.38 (0.09–1.55)	0.33 (0.08–1.35)	0.33 (0.08–1.36)	0.41 (0.10–1.72)
4 cups/d	0.70 (0.17–2.87)	0.59 (0.14–2.45)	0.54 (0.13–2.27)	0.65 (0.15–2.71)
≥ 5 cups/d	1.12 (0.35–3.60)	0.99 (0.31–3.20)	0.84 (0.26–2.73)	1.06 (0.32–3.48)
Any amount	0.57 (0.34–0.97)	0.52 (0.30–0.88)	0.50 (0.28–0.88)	0.62 (0.35–1.11)
Any amount (excluding cases within first 5 years)	0.49 (0.25–0.99)	0.44 (0.22–0.88)	0.51 (0.24–1.08)	0.60 (0.27–1.30)
**Death from CLD**				
No coffee	1 (ref.)	1 (ref.)	1 (ref.)	1 (ref.)
0.5 cups/d	0.23 (0.06–0.95)	0.23 (0.06–0.92)	0.21 (0.05–0.86)	0.23 (0.06–0.96)
1 cup/d	0.26 (0.12–0.60)	0.25 (0.11–0.58)	0.22 (0.10–0.51)	0.27 (0.12–0.63)
2 cups/d	0.46 (0.24–0.88)	0.41 (0.22–0.80)	0.34 (0.17–0.65)	0.38 (0.19–0.75)
3 cups/d	0.26 (0.08–0.81)	0.23 (0.07–0.71)	0.17 (0.05–0.55)	0.22 (0.07–0.69)
4 cups/d	0.63 (0.23–1.71)	0.54 (0.20–1.47)	0.40 (0.15–1.09)	0.50 (0.18–1.36)
≥ 5 cups/d	1.35 (0.66–2.78)	1.15 (0.56–2.37)	0.70 (0.32–1.51)	0.85 (0.39–1.85)
Any amount	0.43 (0.29–0.63)	0.39 (0.26–0.58)	0.31 (0.21–0.47)	0.37 (0.24–0.56)
Any amount (excluding cases within first 5 years)	0.47 (0.29–0.76)	0.43 (0.26–0.70)	0.36 (0.22–0.61)	0.42 (0.25–0.71)

Abbreviations: *CLD* (chronic liver disease), *HCC* (hepatocellular carcinoma). ^1^unadjusted, ^2^adjusted for age and sex, ^3^as for ^2^ and additionally adjusted for deprivation (Townsend score), smoking status (never, current or previous), diabetes (yes or no), ethnicity (white or other ethnicity), alcohol frequency (less than weekly, once or twice a week, three or four times a week, and daily or almost daily) and BMI, ^4^as for ^3^ but adjusted for weekly alcohol units instead of alcohol frequency

## Discussion

This cohort study investigated associations of coffee consumption with incident CLD, incident CLD or steatosis, incident HCC and death from CLD among nearly half a million participants, of whom 78% were regular coffee drinkers (median consumption of two cups each day). During a median follow-up of 10.7 years, there were 3600 cases of incident CLD, 5439 cases of incident CLD or steatosis, 184 cases of incident HCC and 301 deaths from CLD. Compared to non-coffee drinkers, coffee drinkers (all types and amounts combined) had 21, 20 and 49% reduced risks of incident CLD, incident CLD or steatosis and death from CLD, respectively. The maximal protective effect was seen at around 3–4 cups each day. The findings were robust to excluding events in the first 5 years. Drinkers of decaffeinated, instant and ground coffee (including espresso) also had lower risks of incident CLD, incident CLD or steatosis, death from CLD and, to a lesser extent, HCC, with ground coffee (including espresso) having the largest effect.

This study agrees with previous cohort studies that generally report inverse associations between coffee consumption and CLD outcomes, including deranged liver enzymes [18], fibrosis [19], cirrhosis [7] and HCC [6]. The protective effects of coffee have been observed in different CLD aetiologies, such as ALD [7], NAFLD [20] and chronic viral hepatitis [21]. Previous studies also report a dose-response relationship up to 5 cups each day [6] but there are limited data above this range. The inverse association between coffee and HCC was weaker in this study compared to previously reported estimates [6, 22, 23]. This was likely because of low power from a small number of HCC cases and a shorter follow-up time compared to other studies (e.g. 10.7 years compared to the 18–20 year follow-up times used by Setiawan et al. and Lai et al. [9, 23]). There may also have been a lower proportion of ground coffee drinkers, which may be more protective than other coffee types (see below).

The association between coffee consumption and NAFLD was investigated in a recent Mendelian randomisation study [24]. In that study, lifetime exposure to coffee was estimated using genetic variants, which are fixed before birth and not affected by confounders. While there was an inverse association between coffee and NAFLD, it did not reach statistical significance (odds ratio 0.76; 95% CI 0.51–1.14). The lack of significance may have been because the genetic variants used (four single nucleotide polymorphisms) only explained a small proportion of the variability of actual coffee consumption.

This study is the first, to our knowledge, to directly investigate the effect of different coffee types on CLD outcomes in a single large cohort. There are few reports in the literature about specific coffee types. A small study in France found that filtered ground coffee but not espresso was associated with a reduced risk of fibrosis in obese women with NAFLD [25]. Indirect conclusions about other coffee types can be inferred from studies in countries where drinking preferences differ. For example, in Finland and Japan instant coffee is the most popular type, and inverse associations with CLD outcomes have been reported in both those countries [23, 26].

In the present study, decaffeinated coffee consumption was associated with similar reductions in risks of incident CLD and incident CLD or steatosis to all coffee types combined and a larger reduction in risk of death from CLD (63% compared to 49%). A meta-analysis of three studies reported inverse associations between decaffeinated coffee and HCC, though smaller in magnitude compared to caffeinated coffee [6]. A cohort study in the United States reported a lower risk of death from CLD among drinkers of two cups of decaffeinated coffee each day compared to none, even after adjustment for caffeinated coffee intake [9]. The observation of a protective effect of decaffeinated coffee is highly relevant to the development of a coffee-based intervention for preventing CLD onset or progression. Caffeine intolerance may limit increases in coffee consumption, and thus decaffeinated coffee may be a preferable alternative. Given its well know safety profile and cheap cost, coffee has potential as widely accessible lifestyle intervention, even in low to middle-income countries.

Strengths of this study include the large cohort size, small numbers of exclusions (~ 2% in the analysis adjusted for alcohol frequency), the wide range of baseline data on key confounders with which to make adjustments in multivariate analyses, and the detailed data on type of coffee consumption.

A limitation is the observational cohort design, which cannot infer causation. There was a single timepoint of coffee consumption, and volumes and preferred types may have changed over the follow-up period. Cup sizes may also have varied. However, misclassification of coffee consumption would have pushed the effect size towards null and not explain our results. There was also no data on ex-coffee drinkers, which may be relevant to reverse causation, as is discussed below. In addition, there may have been differences in chemical composition between coffees within the same type (e.g. due to different processes for decaffeination).

The risk of confounding was reduced by making adjustments for baseline covariates, but these may have been assessed inaccurately or changed during follow-up. In relation to alcohol consumption, a key CLD risk factor, the assumptions used to convert drinks into units, such as the volume and alcohol content of each beverage, may have been inaccurate. In addition, imputation of alcohol units was not used for missing data. The HRs adjusted for weekly units were similar to those adjusted for alcohol consumption frequency. While there may have been some residual confounding from alcohol, it is unlikely that this alone is responsible for the findings of this study.

Bias may have been introduced from incomplete adjustment for socio-economic status using Townsend deprivation index scores rather than individual level socioeconomic variables (e.g. education) [12]. This would have exaggerated the effect sizes because deprivation was associated with CLD and non-coffee drinkers were more deprived than coffee drinkers.

There were small differences in proportions of ethnicities in the non-coffee drinking reference group compared to the coffee drinking groups. Prevalence and aetiology of CLD is known to vary between ethnic groups [27], and this would have introduced bias if not fully accounted for by binary adjustment for white or other ethnicity. The results were also not adjusted for waist circumference, which may be related to metabolic syndrome independently of BMI, or prediabetes. Other CLD risk factors not adjusted for included chronic infection with hepatitis B or C, though prevalence of these conditions was likely low in this UK volunteer cohort.

Some cases and non-cases may have been misclassified because of inaccuracies in the coded data used to ascertain outcomes. Participants with an associated ICD-10 code K76.0 in the absence of other additional codes identifying CLD were assumed to have simple steatosis rather than more established forms of NAFLD (e.g. nonalcoholic steatohepatitis, which corresponds to K75.81). While this assumption may have led to under-ascertainment of CLD cases, the risk of bias was likely small given the similar associations of all coffee types with CLD and CLD or steatosis.

Reverse causation was addressed by excluding diagnosed CLD at baseline. The presence of undiagnosed cases might have exaggerated the effect sizes if CLD resulted in coffee intolerance, and it was not possible to identify previous coffee drinkers in our non-coffee drinker category. However, the protective effect of coffee was robust to exclusion of CLD outcomes in the first five years of follow-up, suggesting that any effect of reverse causation was small.

It was not possible to fully assess the effects of very high levels of coffee consumption as the numbers of events in these categories were low. The risk reduction for ≥5 cups each day was generally smaller than for 3 or 4 cups each day, though still protective compared to no coffee, indicating that there is likely a level beyond which increasing coffee consumption confers no further benefit. Dose-response meta-analyses report protective effects up to around 5 cups each day with increasing uncertainty thereafter [6, 7].

The results of this study may be limited by the demographics of the voluntary UK Biobank Cohort, who were predominantly white and likely over-representative of those from higher socio-economic groups. As such, the findings may not generalise to populations with a very different ethnic and socio-economic composition.

Confidence in the veracity of the observed inverse associations of coffee consumption with CLD outcomes is increased by several factors. These include the magnitude and significance of the effect sizes and the presence of a dose response. In addition, residual confounding was likely to have led to underestimation of the effect sizes given that the adjusted HRs were generally larger in magnitude and significance than the unadjusted HRs, indicating coffee drinkers had a greater overall burden of known CLD risk factors.

There is biological plausibility of a protective effect of coffee against CLD outcomes. Caffeine is a non-selective antagonist of the A2aA receptor, activation of which stimulates collagen production by hepatic stellate cells, the primary mediators of fibrosis [28, 29]. However, in the present study as well as in previous studies [6] decaffeinated coffee was also protective. Alternative active ingredients in coffee may include chlorogenic acid, kahweol and cafestol, which protect against liver fibrosis in animal studies [30, 31]. Kahweol and cafestol are present in highest concentrations in ground coffee, which was most protective. Given the protective effects of the different coffee types with varying composition, there may be a complex relationship involving more than one active ingredient.

## Conclusions

This study provides evidence of a protective effect of all types of coffee (including decaffeinated) against CLD outcomes. These findings are significant given the paucity of effective preventative and treatment strategies for CLD, especially in low to medium income countries, where the burden of CLD is highest. Further work is now needed to replicate these findings using more robust methods, including Mendelian randomisation with a more powerful set of genetic variants to estimate coffee consumption than available previously. Randomised trials should then investigate the efficacy of a coffee-based intervention in those at risk of CLD or its complications.

## Supplementary Information

**Additional file 1.**

## Data Availability

Data for this study are available on application to UK Biobank (https://www.ukbiobank.ac.uk/).

## Abbreviations

ALD: Alcohol-related liver disease
BMI: Body mass index
CI: Confidence intervals
CLD: Chronic liver disease
HR: Hazard ratio
HCC: Hepatocellular carcinoma
IQR: Interquartile range
NAFLD: Non-alcoholic fatty liver disease
n: Number
PH: Proportional hazards
STROBE: Strengthening the reporting of observational studies in epidemiology
