# Supplementary material for: All coffee types decrease the risk of adverse clinical outcomes in chronic liver disease: a UK Biobank study
Source: BMC Public Health. 2021 Jun 22;21:970. doi: 10.1186/s12889-021-10991-7 (PMC8218514; doi:10.1186/s12889-021-10991-7)
Supplement: Supplementary file 1 — Additional file 1. [file 12889_2021_10991_MOESM1_ESM.pdf]

All coffee types decrease the risk of adverse clinical outcomes in chronic liver disease: A UK Biobank study

Oliver J. Kennedy[1], Jonathan A. Fallowfield[2], Robin Poole[1], Peter C. Hayes[2], Julie Parkes[1] and Paul J. Roderick[1]

1. Primary Care & Population Sciences Faculty of Medicine, University of Southampton, Southampton, SO17 1BJ, UK

2. University of Edinburgh Centre for Inflammation Research, Queen's Medical Research Institute, Edinburgh Bioquarter, Edinburgh, EH16 4TJ, UK

Supplementary information

Figures

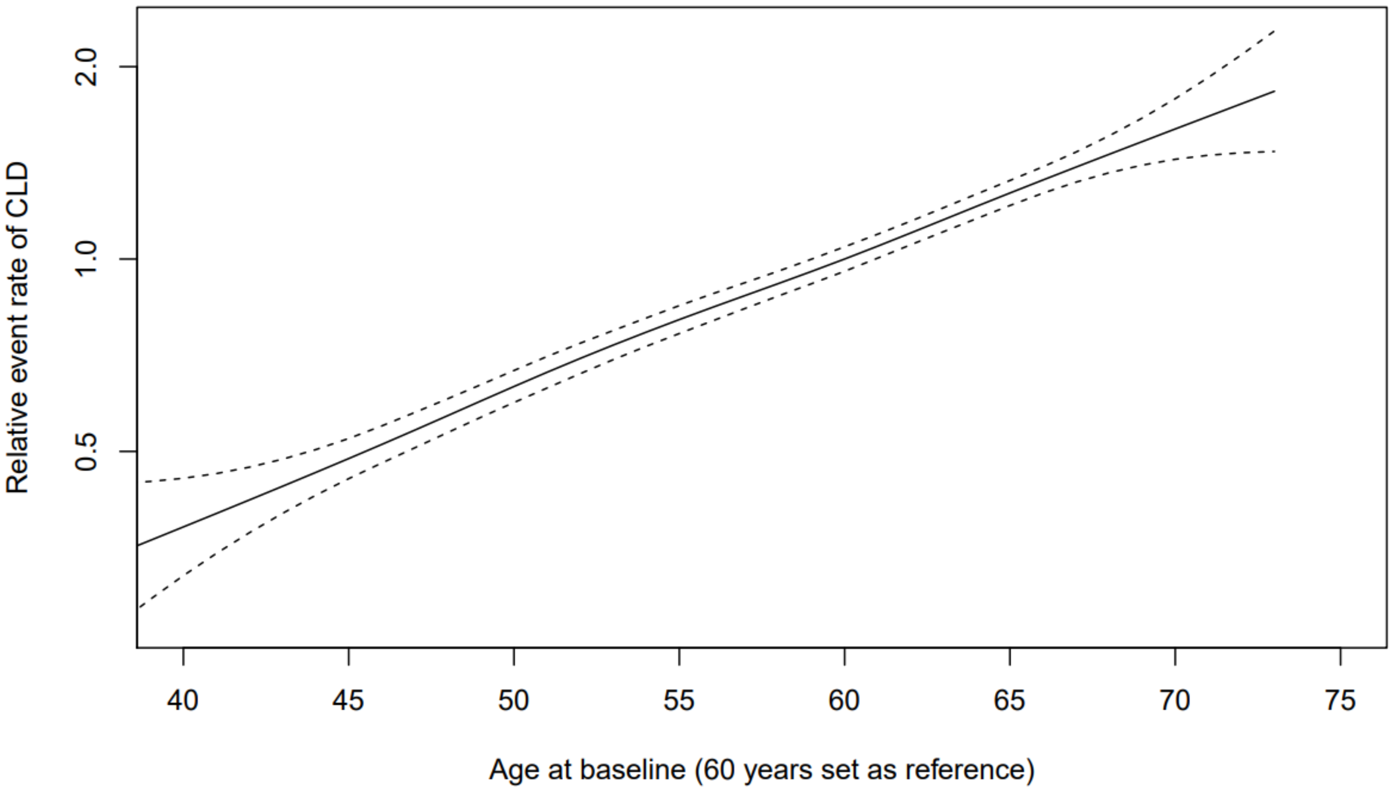

Supplementary figure 1. A graph showing the association of age with the relative event rate of chronic liver disease as modelled using a penalised spline.

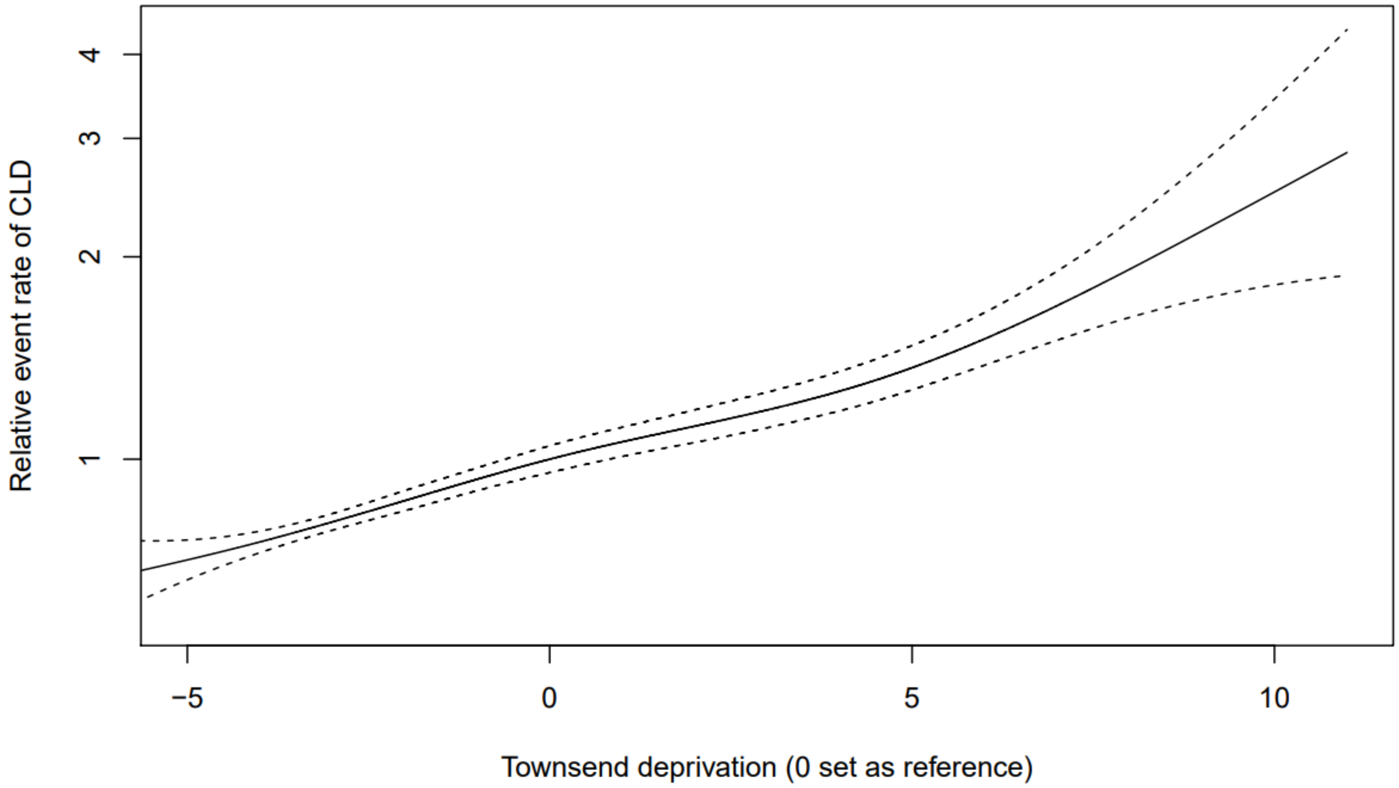

Supplementary figure 2. A graph showing the association of Townsend deprivation index with the relative event rate of chronic liver disease as modelled using a penalised spline.

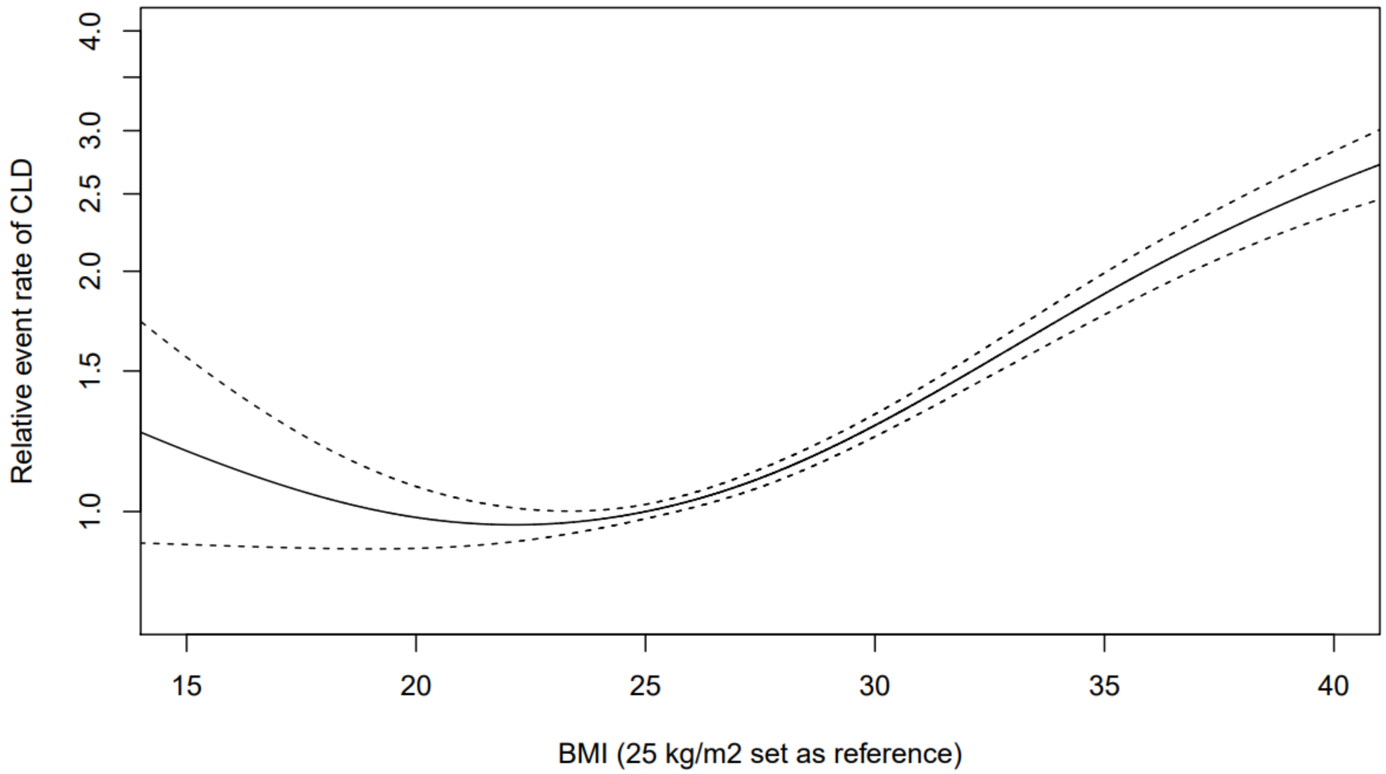

Supplementary figure 3. A graph showing the association of body mass index with the relative event rate of chronic liver disease as modelled using a penalised spline.

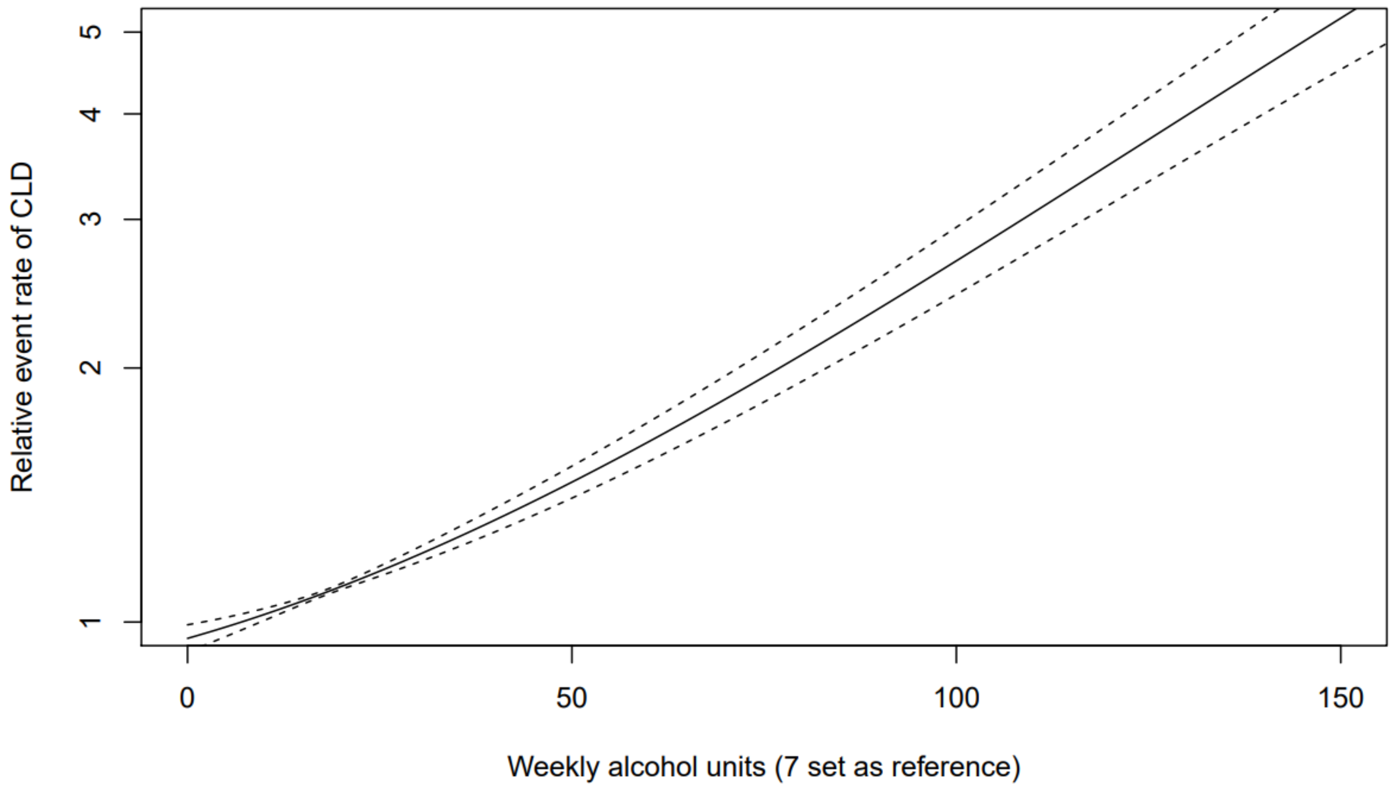

Supplementary figure 4. A graph showing the association of weekly alcohol units with the relative event rate of chronic liver disease as modelled using a penalised spline.

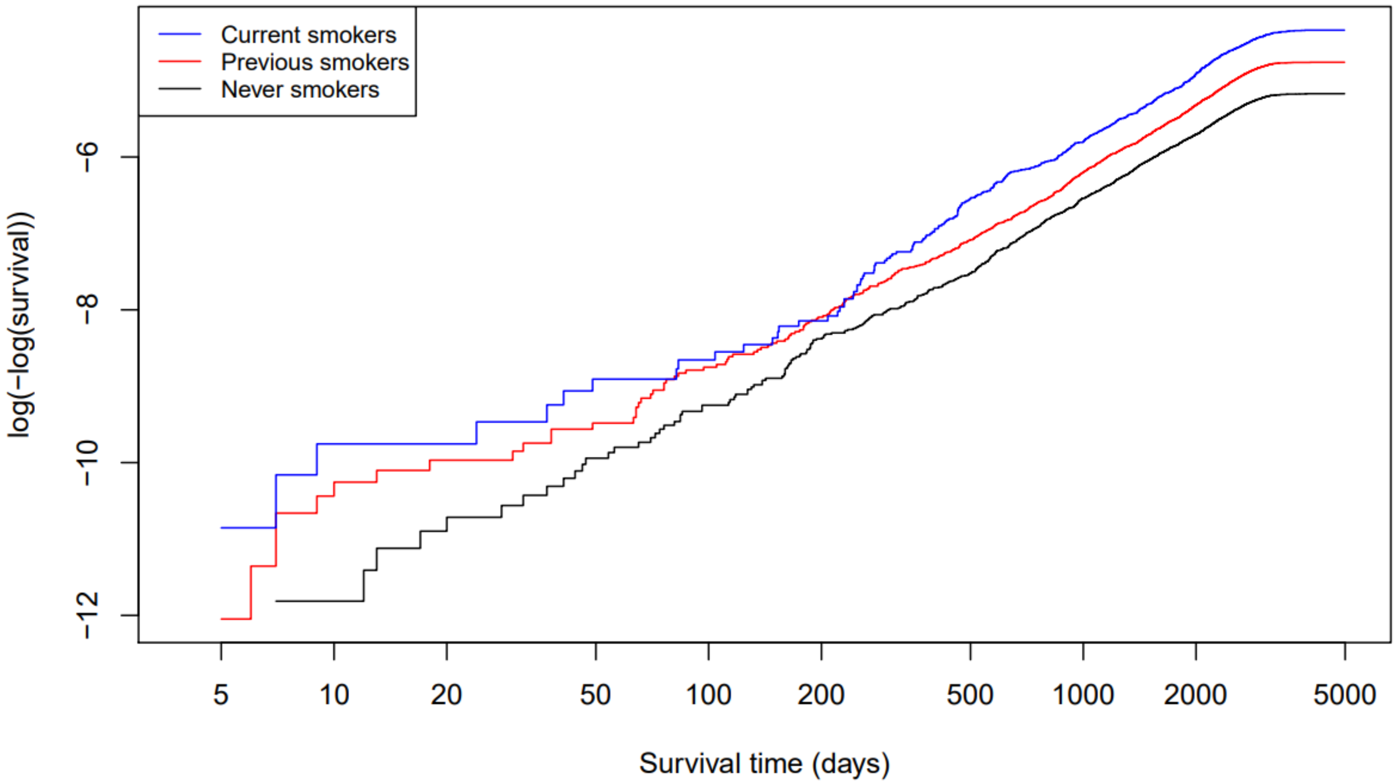

Supplementary figure 5. A log(-log) plot for chronic liver disease and smoking status.

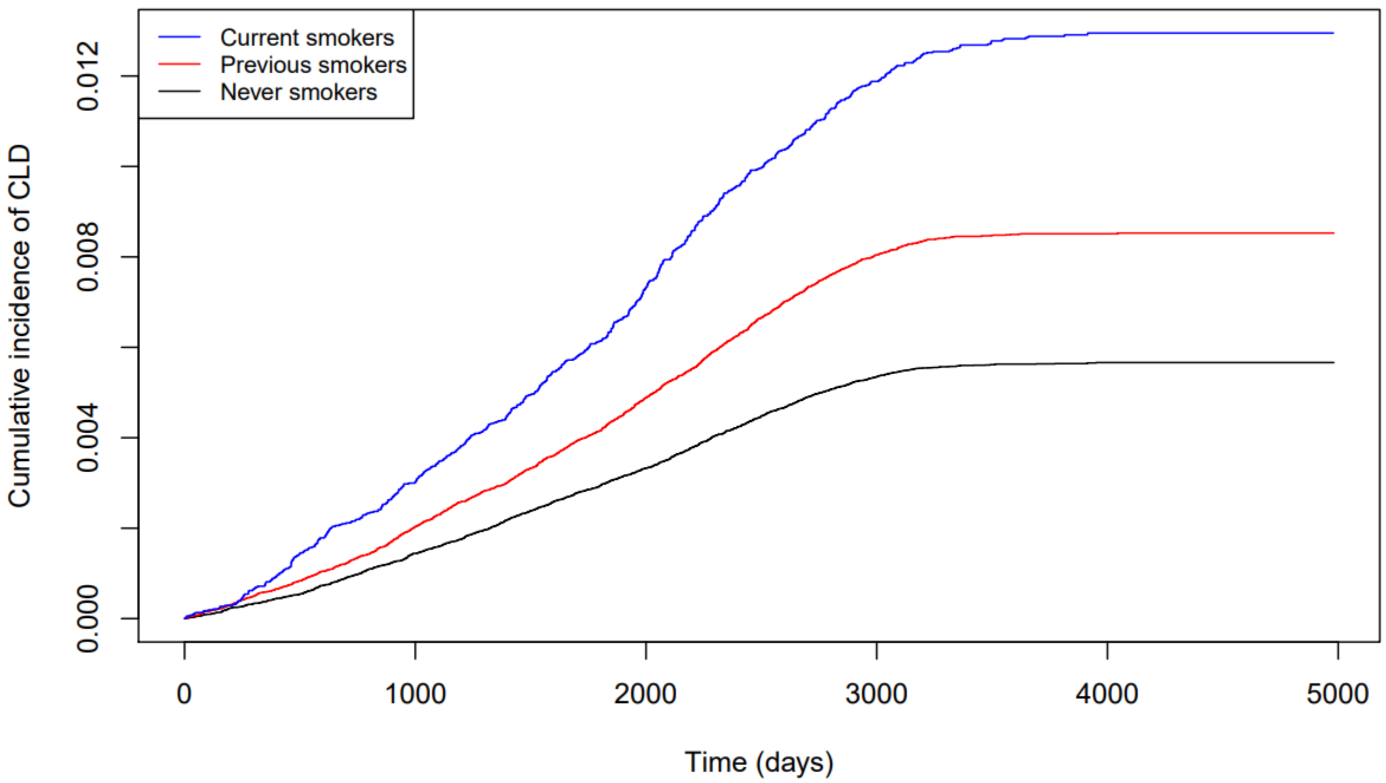

Supplementary figure 6. A plot showing the cumulative incidence of chronic liver disease according to smoking status

Tables

Supplementary table 1. UK Biobank participants were asked to specify amounts of different alcohol types consumed each week or month. This table details the assumptions used to calculate "unit" equivalents (1 unit is equal to 10 mL of pure ethanol) for each drink.

**Drink type**	**Units equivalent**
Measure of spirit	1
Glass of fortified wine	1·25 (i.e. 12 glasses in 750 mL 20% vol bottle)
Other alcoholic drink	1·5 (i.e. one Smirnoff Ice)
Pint of beer or cider	2·5 (i.e. 568 mL of 4% vol)
Glass of white wine / Champaign	1·75 (i.e. 6 glasses in 750 mL 14% vol bottle)
Glass of red wine	1·75 (i.e. 6 glasses in 750 mL 14% vol bottle)

Supplementary table 2. Baseline characteristics of the UK Biobank cohort according to the availability of baseline weekly alcohol units.

	**Weekly alcohol units available**	**No weekly alcohol units available**
n	415961	80854
Median age at baseline (IQR)	58 (13)	57 (14)
Sex (% female)	52·3	65·7
**Ethnicity (%)**		
White	94·4	92·1
Other ethnicity	5·3	7·4
Unknown	0·3	0·6
Median Townsend deprivation score	-2·21	-1·79
Median coffee consumption (cups/d)	2	1
**Alcohol frequency (%)**		
Never	9·5	0
Special occasions only	4·9	45·6
One to three times a month	4·5	44·9
Once or twice a week	30·2	3·2
Three or four times a week	27·1	2·3
Daily or almost daily	23·8	2·1
Unknown	0	0·7
**Diabetes**		
Yes	4·9	6·7
No	94·8	91·6
Unknown	0·3	1·7
**Median BMI (IQR)**	26·6 (5·6)	27·4 (6·7)
**Smoking**		
Never	53·8	58·2
Previous	35·7	27·9

Current<br>Unknown	10·1<br>0·3	12·1<br>1·8
CLD cases (%)	2950 (0.71)	670 (0.83)
CLD or steatosis cases (%)	4401 (1.06)	1070 (1.32)
HCC cases (%)	154 (0.04)	30 (0.04)
CLD deaths (%)	266 (0.06)	36 (0.04)

Abbreviations: IQR (interquartile range), BMI (body mass index), CLD (chronic liver disease), HCC (hepatocellular carcinoma)

Supplementary tables 3a, 3b and 3c. Conditions corresponding to ICD-10 codes, ICD-9 codes and self-reported diagnoses that were used to exclude participants at baseline who may have had CLD. The ICD codes were used to identify condition recorded in hospital and cancer records before recruitment. The self-reported diagnoses were disclosed during interview at a baseline assessment centre visit.

Table 3a

**ICD-10 codes**
B18·0 Chronic viral hepatitis B with delta-agent
B18·1 Chronic viral hepatitis B without delta-agent
B18·2 Chronic viral hepatitis C
B18·8 Other chronic viral hepatitis
B18·9 Chronic viral hepatitis, unspecified
B19·0 Unspecified viral hepatitis with coma
B19·9 Unspecified viral hepatitis without coma
B58·1 Toxoplasma hepatitis
B25·1 Cytomegaloviral hepatitis
C22·0 Liver cell carcinoma
C22·1 Intrahepatic bile duct carcinoma
C22·2 Hepatoblastoma
C22·3 Angiosarcoma of liver
C22·4 Other sarcomas of liver
C22·7 Other specified carcinomas of liver
C22·9 Liver, unspecified (malignancy)
C78·7 Secondary malignant neoplasm of liver
D01·5 Liver, gallbladder and bile ducts (carcinoma)
D37·6 Liver, gallbladder and bile ducts (neoplasm of uncertain significance)
I85·0 Oesophageal varices with bleeding
I85·9 Oesophageal varices without bleeding
I86·4 Gastric varices
I98·2 Oesophageal varices in diseases classified elsewhere
I98·3 Oesophageal varices with bleeding in diseases classified elsewhere
K70·0 Alcoholic fatty liver
K70·1 Alcoholic hepatitis
K70·2 Alcoholic fibrosis and sclerosis of liver
K70·3 Alcoholic cirrhosis of liver
K70·4 Alcoholic hepatic failure
K70·9 Alcoholic liver disease, unspecified
K71·0 Toxic liver disease with cholestasis
K71·1 Toxic liver disease with hepatic necrosis
K71·2 Toxic liver disease with acute hepatitis
K71·3 Toxic liver disease with chronic persistent hepatitis
K71·4 Toxic liver disease with chronic lobular hepatitis
K71·5 Toxic liver disease with chronic active hepatitis
K71·6 Toxic liver disease with hepatitis, not elsewhere classified
K71·7 Toxic liver disease with fibrosis and cirrhosis of liver
K71·8 Toxic liver disease with other disorders of liver
K71·9 Toxic liver disease, unspecified
K72·0 Acute and subacute hepatic failure
K72·1 Chronic hepatic failure
K72·9 Hepatic failure, unspecified
K73·0 Chronic persistent hepatitis, not elsewhere classified
K73·1 Chronic lobular hepatitis, not elsewhere classified
K73·2 Chronic active hepatitis, not elsewhere classified
K73·8 Other chronic hepatitis, not elsewhere classified
K73·9 Chronic hepatitis, unspecified
K74·0 Hepatic fibrosis
K74·1 Hepatic sclerosis
K74·2 Hepatic fibrosis with hepatic sclerosis
K74·3 Primary biliary cirrhosis
K74·4 Secondary biliary cirrhosis
K74·5 Biliary cirrhosis, unspecified
K74·6 Other and unspecified cirrhosis of liver
K75·0 Abscess of liver
K75·1 Phlebitis of portal vein
K75·2 Nonspecific reactive hepatitis
K75·3 Granulomatous hepatitis, not elsewhere classified
K75·4 Autoimmune hepatitis
K75·8 Other specified inflammatory liver diseases
K75·9 Inflammatory liver disease, unspecified

K76·0 Fatty (change of) liver, not elsewhere classified
K76·1 Chronic passive congestion of liver
K76·2 Central haemorrhagic necrosis of liver
K76·3 Infarction of liver
K76·4 Peliosis hepatis
K76·5 Hepatic veno-occlusive disease
K76·6 Portal hypertension
K76·7 Hepatorenal syndrome
K76·8 Other specified diseases of liver
K76·9 Liver disease, unspecified
K77·0 Liver disorders in infectious and parasitic diseases classified elsewhere
K77·8 Liver disorders in other diseases classified elsewhere
O26·6 Liver disorders in pregnancy, childbirth and the puerperium
Q44·6 Cystic disease of liver
Q44·7 Other congenital malformations of liver
T86·4 Liver transplant failure and rejection
Z52·6 Liver donor
Z94·4 Liver transplant status
P35·3 Congenital viral hepatiti
R18 Ascites
Z22·5 Carrier of viral hepatitis

Abbreviations: ICD-10 (International Classification of Diseases 10th Revision)

Table 3b

**ICD-9 codes**
0702 Viral hepatitis b with hepatic coma
0703 Viral hepatitis b without mention of hepatic coma
0704 Other specified viral hepatitis with hepatic coma
0705 Other specified viral hepatitis without mention of hepatic coma
0706 Unspecified viral hepatitis with hepatic coma
0709 Unspecified viral hepatitis without mention of hepatic coma
1550 Malignant neoplasm of liver, primary
2308 Carcinoma in situ of liver and biliary system
2353 Neoplasm of uncertain behaviour of liver and biliary passages
4560 Oesophageal varices with bleeding
4561 Oesophageal varices without mention of bleeding
4562 Oesophageal varices in cirrhosis of liver
5710 Alcoholic fatty liver
5712 Alcoholic cirrhosis of liver
5713 Alcoholic liver damage, unspecified
5714 Chronic hepatitis
57150 Cirrhosis of liver without mention of alcohol (congestive)
57151 Cirrhosis of liver without mention of alcohol (postnecrotic)
57152 Cirrhosis of liver without mention of alcohol (childhood function)
57158 Portal fibrosis without cirrhosis of liver without mention of alcohol
57159 Cirrhosis of liver without mention of alcohol (other and unspecified)
5716 Biliary cirrhosis
5718 Other chronic nonalcoholic liver disease (only included in incident CLD including steatosis outcome)
5719 Unspecified chronic liver disease without mention of alcohol
5723 Portal hypertension
5724 Hepatorenal syndrome
5728 Other sequelae of chronic liver disease
5731 Hepatitis in viral diseases classified elsewhere
5732 Hepatitis in other infectious diseases classified elsewhere
5733 Hepatitis unspecified
5734 Hepatic infarction
7895 Ascites
V026 Carrier or suspected carrier of viral hepatitis
27103 Glycogenosis (associated with hepatic cirrhosis)
27761 Other deficiencies of circulating enzymes (alpha-1-antitrypsin hepatitis)
75161 Cystic or fibrocystic disease of liver
V427 Liver replaced by transplant
5709 Acute and subacute necrosis of liver
5728 Other sequelae of chronic liver disease
5730 Chronic passive congestion of liver
6467 Liver disorders in pregnancy
7516 Anomalies of gallbladder, bile ducts and liver
75160 Absence or agenesis of liver, total or part
75162 Other anomalies of liver
9641 Poisoning by liver preparations and other antianaemic agents

Abbreviation: ICD-9 International Classification of Diseases 9th Revision),

Table 3c

**Self-reported conditions**
alcoholic liver disease / alcoholic cirrhosis
liver/biliary/pancreas problem
liver failure/cirrhosis
oesophageal varices
infective/viral hepatitis
hepatitis b
hepatitis c
non-infective hepatitis
liver/hepatocellular cancer
Self-reported conditions:
alcoholic liver disease / alcoholic cirrhosis
liver/biliary/pancreas problem
liver failure/cirrhosis
oesophageal varices
infective/viral hepatitis
hepatitis b
hepatitis c

Supplementary table 4. Baseline characteristics of the UK Biobank cohort according to the availability of baseline coffee consumption.

	**Coffee consumption available**	**No coffee consumption available**
N	494585	2230
Median age (years) at baseline (IQR)	58 (13)	56 (15)
Sex (% female)	54·5	48·3
**Ethnicity (%)**		
White	94·3	34·9
Other ethnicity	5·4	58·7
Unknown	0·3	6·4
Median Townsend deprivation score	-2·15	1·43
**Alcohol frequency (%)**		
Never	7·9	16·2
Special occasions only	11·5	10·8
One to three times a month	11·1	4·3
Once or twice a week	25·8	10·6
Three or four times a week	23·1	6·1
Daily or almost daily	20·4	4·1
Unknown	0·1	8·2
Median units each week (IQR)	15·8 (24·5)	0 (11·9)
**Diabetes (%)**		
Yes	5·2	7·4
No	94·5	44·8
Unknown	0·3	47·8
**Median BMI in kg/m^2 (IQR)**	26·7 (5·8)	27·7 (6·5)
**Smoking (%)**		
Never	54·7	28·9
Previous	34·5	11·9
Current	10·5	11·2
Unknown	0·4	48·0
CLD cases (%)	3600 (0.73)	20 (0.90)
CLD or steatosis cases (%)	5439 (1.10)	32 (1.43)
HCC cases (%)	184 (0.04)	0 (0.00)
CLD deaths (%)	301 (0.06)	1 (0.04)

Abbreviations: IQR (interquartile range), BMI (body mass index), CLD (chronic liver disease), HCC (hepatocellular carcinoma)

Supplementary table 5. Baseline characteristics of the UK Biobank cohort and distribution of incident chronic liver disease, incident chronic liver disease or steatosis, death from chronic liver disease and incident hepatocellular carcinoma according to type of coffee consumption.

**Coffee type**	**Decaffeinated**	**Instant**	**Ground**
N	73644	212586	86987
Median age (years) at baseline (IQR)	59 (12)	58 (13)	57 (13)
Sex (% female)	63·1	50·5	51·7
**Ethnicity (%)**			
White	96·0	95·6	96·0
Other ethnicity	3·8	4·1	3·5
Unknown	0·3	0·3	0·5
Median Townsend deprivation score	-2·54	-2·26	-2·07
**Alcohol frequency (%)**			
Never	8·1	5·9	4·0
Special occasions only	13·4	10·6	6·0
One to three times a month	12·9	11·0	8·1
Once or twice a week	27·9	27·2	22·1
Three or four times a week	22·2	24·2	28·8
Daily or almost daily	15·4	21·1	30·9

Unknown	0·1	0·1	0
Median units each week (IQR)	12·8 (20)	17·2 (25·2)	20·2 (25·5)
**Diabetes (%)**			
Yes	5·0	5·4	3·9
No	94·6	94·3	96·0
Unknown	0·3	0·3	0·2
**Median BMI in kg/m^2 (IQR)**	26·7 (5·8)	27·0 (5·7)	26·0 (5·4)
**Smoking (%)**			
Never	59·5	51·3	53·3
Previous	33·4	35·7	37·1
Current	6·7	12·6	9·3
Unknown	0·4	0·4	0·2
CLD cases (%)	481 (0.65)	1625 (0.76)	477 (0.55)
CLD or steatosis cases (%)	759 (1.03)	2426 (1.14)	692 (0.80)
HCC cases (%)	29 (0.04)	90 (0.04)	20 (0.02)
CLD deaths (%)	23 (0.03)	140 (0.07)	33 (0.04)

Supplementary table 6. Baseline characteristics of the UK Biobank cohort and distribution of incident chronic liver disease, incident chronic liver disease or steatosis, death from chronic liver disease and incident hepatocellular carcinoma according to decaffeinated coffee consumption.

**Coffee (cups/d)**	**0**	**Half**	**1**	**2**	**3**	**4**	**≥5**
N	109767	6807	19406	17397	11169	8094	10771
Median age (years) at baseline (IQR)	56 (13)	58 (13)	60 (12)	60 (12)	60 (12)	59 (12)	58 (12)
Sex (% female)	58·3	66·0	65·5	63·3	62·6	60·6	59·3
**Ethnicity (%)**							
White	89·7	92·8	94·0	96·2	97·5	98·1	97·9
Other ethnicity	9·9	6·9	5·7	3·6	2·3	1·7	1·8
Unknown	0·4	0·2	0·3	0·2	0·2	0·2	0·3
Median Townsend deprivation score	-1·70	-2·48	-2·48	-2·6	-2·68	-2·57	-2·4
**Alcohol frequency (%)**							
Never	14·4	8·6	8·8	7·5	7·0	7·4	9·0
Special occasions only	15·9	15·7	14·3	12·6	12·1	12·0	14·3
One to three times a month	12·6	14·4	13·5	12·2	12·3	12·4	12·9
Once or twice a week	24·9	27·0	28·5	28·9	27·1	27·7	26·9
Three or four times a week	17·6	20·4	21·1	22·8	24·3	23·7	21·4
Daily or almost daily	14·5	13·9	13·7	16·0	17·2	16·9	15·4
Unknown	0·1	0·1	0·1	0·1	0	0	0·1
Mean weekly units (IQR)	11 (24·3)	11 (19·6)	11·5 (19·2)	13 (19·5)	13·8 (20·5)	14 (21·2)	13·5 (22·3)
**Diabetes (%)**							
Yes	5·7	4·1	5·0	4·9	5·3	5·0	5·7
No	93·9	95·6	94·6	94·8	94·5	94·7	93·9
Unknown	0·4	0·4	0·4	0·3	0·2	0·3	0·3
**Median BMI in kg/m^2 (IQR)**	26·8 (6·1)	26·0 (5·7)	26·3 (5·6)	26·5 (5·5)	26·8 (5·7)	27·2 (5·8)	27·6 (6·2)
**Smoking (%)**							
Never	58·6	64·9	63·4	61·2	59·5	55·8	49·2
Previous	31·3	29·5	31·6	32·6	34	36·1	38·1
Current	9·7	5·2	4·8	5·8	6·1	7·7	12·3
Unknown	0·4	0·4	0·3	0·4	0·4	0·4	0·4
**CLD cases (n)**							
Total (%)	927 (0.84)	49 (0.72)	124 (0.64)	115 (0.66)	70 (0.63)	55 (0.68)	68 (0.63)
First 5 years excluded (%)	449 (0.41)	134 (1.97)	329 (1.70)	320 (1.84)	185 (1.66)	122 (1.51)	213 (1.98)
**CLD or steatosis cases (n)**							
Total (%)	1412 (1.29)	70 (1.03)	189 (0.97)	179 (1.03)	108 (0.97)	83 (1.03)	130 (1.21)
First 5 years excluded (%)	724 (0.66)	41 (0.60)	97 (0.50)	96 (0.55)	54 (0.48)	47 (0.58)	73 (0.68)
**HCC cases (n)**							
Total (%)	44 (0.04)	0 (0.00)	9 (0.05)	8 (0.05)	6 (0.05)	4 (0.05)	2 (0.02)
First 5 years excluded (%)	28 (0.03)	0 (0.00)	6 (0.03)	6(0.03)	4 (0.04)	4 (0.05)	0 (0.00)
**CLD deaths**							
Total (%)	97 (0.09)	3 (<0.01)	5 (<0.01)	6 (<0.01)	3 (<0.01)	4 (<0.01)	2 (<0.01)
First 5 years excluded (%)	59 (0.05)	0 (0.04)	3 (0.03)	5 (0.03)	1 (0.03)	2 (0.05)	1 (0.02)

Abbreviations: N (number), BMI (body mass index), IQR (interquartile range), CLD (chronic liver disease), HCC (hepatocellular carcinoma)

Supplementary table 7. Baseline characteristics of the UK Biobank cohort and distribution of incident chronic liver disease, incident chronic liver disease or steatosis, death from chronic liver disease and incident hepatocellular carcinoma according to instant coffee consumption.

**Coffee (cups/d)**	**0**	**Half**	**1**	**2**	**3**	**4**	**≥5**
N	109767	17152	50464	48606	34789	25383	36192
Median age (years) at baseline (IQR)	56 (13)	58 (12)	59 (12)	59 (13)	58 (13)	58 (13)	56 (13)
Sex (% female)	58·3	51·0	54·1	51·7	50·4	48·3	45·4
**Ethnicity (%)**							
White	89·7	92·2	93·3	95·5	96·9	97·5	97·8
Other ethnicity	9·9	7·5	6·4	4·2	2·8	2·2	1·9
Unknown	0·4	0·3	0·3	0·3	0·3	0·3	0·3
Median Townsend deprivation score	-1·70	-2·09	-2·26	-2·34	-2·39	-2·35	-1·99
**Alcohol frequency (%)**							
Never	14·4	7·0	6·4	5·2	4·9	4·9	7·2
Special occasions only	15·9	12·7	11·8	9·5	8·8	9·2	12·0

One to three times a month	12·6	12·7	11·3	10·4	10·2	10·1	11·9
Once or twice a week	24·9	27·4	28·1	27·5	26·7	26·9	25·9
Three or four times a week	17·6	21·9	23·1	25·4	26·1	25·7	22·1
Daily or almost daily	14·5	18·2	19·2	21·9	23·3	23·1	20·7
Unknown	0·1	0·1	0·1	0·1	0·1	0·1	0·1
Mean weekly units (IQR)	11 (24·3)	15 (25·5)	15·2 (24·0)	17·5 (24·5)	18·5 (24·8)	19·5 (26)	18 (28·5)
**Diabetes (%)**							
Yes	5·7	5·6	5·4	5·2	5·3	5·4	5·7
No	93·9	94·1	94·3	94·6	94·5	94·3	94·1
Unknown	0·4	0·3	0·3	0·2	0·2	0·3	0·3
**Median BMI in kg/m^2 (IQR)**	26·8 (6·1)	26·8 (5·7)	26·7 (5·6)	26·9 (5·6)	27·0 (5·6)	27·3 (5·7)	27·6 (5·9)
**Smoking (%)**							
Never	58·6	56·0	56·3	53·8	52·3	48·6	39·8
Previous	31·3	34·1	35·0	36·0	36·4	37·2	35·5
Current	9·7	9·5	8·2	9·8	11·0	13·9	24·3
Unknown	0·4	0·4	0·4	0·4	0·4	0·3	0·4
**CLD cases (n)**							
Total (%)	927 (0.84)	162 (0.94)	401 (0.79)	384 (0.79)	237 (0.68)	171 (0.67)	270 (0.75)
First 5 years excluded (%)	449 (0.41)	72 (0.42)	186 (0.37)	193 (0.40)	115 (0.33)	76 (0.30)	144 (0.40)
**CLD or steatosis cases (n)**							
Total (%)	1412 (1.29)	222 (1.29)	604 (1.20)	564 (1.16)	353 (1.01)	256 (1.01)	427 (1.18)
First 5 years excluded (%)	724 (0.66)	104 (0.61)	302 (0.60)	293 (0.60)	188 (0.54)	120 (0.47)	232 (0.64)
**HCC cases (n)**							
Total (%)	44 (0.04)	8 (0.05)	17 (0.03)	28 (0.06)	16 (0.05)	9 (0.04)	12 (0.03)
First 5 years excluded (%)	28 (0.03)	6 (0.03)	12 (0.02)	12 (0.02)	8 (0.02)	5 (0.02)	8 (0.02)
**CLD deaths**							
Total (%)	97 (<0.01)	15 (<0.01)	38 (<0.01)	39 (<0.01)	22 (<0.01)	8 (<0.01)	18 (<0.01)
First 5 years excluded (%)	59 (0.09)	8 (0.09)	24 (0.08)	27 (0.08)	17 (0.06)	4 (0.03)	13 (0.05)

Abbreviations: N (number), BMI (body mass index), IQR (interquartile range), CLD (chronic liver disease), HCC (hepatocellular carcinoma)

Supplementary table 8. Baseline characteristics of the UK Biobank cohort and distribution of incident chronic liver disease, incident chronic liver disease or steatosis, death from chronic liver disease and incident hepatocellular carcinoma according to ground coffee consumption.

**Coffee (cups/d)**	**0**	**Half**	**1**	**2**	**3**	**4**	**≥5**
N	109767	9651	25608	24644	13235	7162	6687
Median age (years) at baseline (IQR)	56 (13)	56 (13)	57 (13)	58 (13)	57 (13)	57 (13)	56 (13)
Sex (% female)	58·3	55·0	56·4	52·8	48·4	44·3	39·4
**Ethnicity (%)**							
White	89·7	95·1	95·3	96·3	96·5	96·8	96·9
Other ethnicity	9·9	4·4	4·3	3·2	2·9	2·6	2·6
Unknown	0·4	0·5	0·4	0·4	0·6	0·5	0·5
Median Townsend deprivation score	-1·70	-2·11	-2·02	-2·06	-2·13	-2·17	-2·00
**Alcohol frequency (%)**							
Never	14·4	3·8	3·9	3·4	3·7	4·6	6·7
Special occasions only	15·9	7·2	6·4	5·4	5·2	5·7	7·2
One to three times a month	12·6	11·1	8·5	7·2	6·6	7·4	9·4
Once or twice a week	24·9	25·1	23·7	21·4	20·3	19·7	20·9
Three or four times a week	17·6	29·0	28·7	29·3	29·5	29·2	25·5
Daily or almost daily	14·5	23·7	28·8	33·2	34·8	33·4	30·3
Unknown	0·1	0	0	0	0	0	0·1
Mean weekly units (IQR)	11 (24·3)	18·2 (24·5)	19 (23·8)	20·8 (24·8)	21·8 (26)	22·5 (27·5)	22·5 (30)
**Diabetes (%)**							
Yes	5·7	4·0	3·5	3·4	3·7	4·8	5·8
No	93·9	95·8	96·3	96·4	96·1	94·9	94·1
Unknown	0·4	0·2	0·2	0·2	0·2	0·3	0·2
**Median BMI in kg/m^2 (IQR)**	26·8 (6·1)	25·9 (5·5)	25·7 (5·3)	25·9 (5·3)	26·1 (5·2)	26·7 (5·4)	27·2 (5·7)
**Smoking (%)**							
Never	58·6	57·1	55·3	53·8	52	49·9	44·7
Previous	31·3	35·1	37·5	37·6	37·7	37·5	35·9
Current	9·7	7·5	7·0	8·4	10·1	12·3	19·1
Unknown	0·4	0·2	0·2	0·3	0·2	0·3	0·4
**CLD cases (n)**							
Total	927 (0.84)	48 (0.50)	150 (0.59)	138 (0.56)	66 (0.50)	36 (0.50)	39 (0.58)
First 5 years excluded	449 (0.41)	28 (0.29)	63 (0.25)	67 (0.27)	30 (0.23)	15 (0.21)	23 (0.34)
**CLD or steatosis cases (n)**							
Total (%)	1412 (1.29)	67 (0.69)	216 (0.84)	198 (0.80)	90 (0.68)	54 (0.75)	67 (1.00)
First 5 years excluded (%)	724 (0.66)	38 (0.39)	98 (0.38)	99 (0.40)	41 (0.31)	26 (0.36)	41 (0.61)
**HCC cases (n)**							
Total (%)	44 (0.04)	2 (0.02)	7 (0.03)	4 (0.02)	2 (0.02)	2 (0.03)	3 (0.04)
First 5 years excluded (%)	28 (0.03)	1 (0.01)	3 (0.01)	3 (0.01)	1 (0.01)	0 (0.00)	3 (0.04)
**CLD deaths**							
Total (%)	97 (<0.01)	2 (<0.01)	6 (<0.01)	10 (<0.01)	3 (<0.01)	4 (<0.01)	8 (<0.01)
First 5 years excluded (%)	59 (0.09)	1 (0.02)	3 (0.02)	9 (0.04)	1 (0.02)	2 (0.06)	6 (0.12)

Abbreviations: N (number), BMI (body mass index), IQR (interquartile range), CLD (chronic liver disease), HCC (hepatocellular carcinoma)

Supplementary table 9. Age and sex adjusted hazard ratios with 95% confidence intervals for associations of covariates with incident chronic liver disease and death from chronic liver disease.

**Covariate**	**HR of incident CLD**	**HR of death from CLD**

**Townsend deprivation (quartiles)**		
1st	1 (ref.)	1 (ref.)
2nd	1·16 (1·05-1·28)	1·10 (0·75-1·61)
3rd	1·34 (1·21-1·47)	1·59 (1·11-2·27)
4th	1·86 (1·70-2·05)	2·68 (1·93-3·72)
**Ethnicity (%)**		
White	1 (ref.)	1 (ref.)
Other ethnicity	1·35 (0·84-2·17)	1·69 (0·42-6·80)
**Smoking status (%)**		
No	1 (ref.)	1 (ref.)
Previous smoker	1·32 (1·23-1·42)	1·70 (1·29-2·24)
Current smoker	2·31 (2·10-2·53)	4·80 (3·58-6·45)
**Diabetes (%)**		
No	1 (ref.)	1 (ref.)
Yes	2·92 (2·66-3·20)	4·00 (3·03-5·28)
**BMI (kg/m^2)**		
<25	1 (ref.)	1 (ref.)
25-<30	1·02 (0·94-1·11)	0·69 (0·51-0·93)
≥30	1·76 (1·62-1·92)	1·84 (1·39-2·45)
**Weekly alcohol units**		
≤14	1 (ref.)	1 (ref.)
>14-≤35 (women) / >14-≤50	0·90 (0·83-0·98)	0·87 (0·63-1·21)
>35 Women / >50 Men	1·67 (1·51-1·84)	3·90 (2·89-5·26)
**Alcohol frequency**		
Less than weekly	1 (ref.)	1 (ref.)
Once or twice a week	0·73 (0·67-0·80)	0·57 (0·38-0·88)
Three or four times a week	0·73 (0·66-0·80)	0·87 (0·60-1·27)
Daily or almost daily	1·11 (1·02-1·21)	2·72 (2·00-3·69)

Abbreviations: BMI (body mass index), CLD (chronic liver disease), HR (hazard ratio)

Supplementary table 10. Adjusted hazard ratios with 95% confidence intervals for associations of coffee consumption with incident chronic liver disease.

	**HR**	n
Diabetes	0.76 (0.60-0.95)[1]	17280
No diabetes	0.80 (0.72-0.88)[1]	371905
BMI<25 (mg/kg^2)	0.77 (0.65-0.92)[2]	132334
BMI 25-30 (mg/kg^2)	0.78 (0.68-0.90)[2]	168026
BMI >30 (mg/kg^2)	0.82 (0.71-0.95)[2]	88825

Abbreviations: BMI (body mass index), HR (hazard ratio). [1]adjusted for age, sex (male, female), deprivation (Townsend score), smoking status (never, current or previous), ethnicity (white or other ethnicity) and weekly alcohol units and BMI. [2]adjusted for age, sex (male or female), deprivation (Townsend score), smoking status (never, current or previous), diabetes (yes or no), ethnicity (white or other ethnicity) and weekly alcohol units and BMI.
